# Reading and editing the *Pleurodeles waltl* genome reveals novel features of tetrapod regeneration

**DOI:** 10.1038/s41467-017-01964-9

**Published:** 2017-12-22

**Authors:** Ahmed Elewa, Heng Wang, Carlos Talavera-López, Alberto Joven, Gonçalo Brito, Anoop Kumar, L. Shahul Hameed, May Penrad-Mobayed, Zeyu Yao, Neda Zamani, Yamen Abbas, Ilgar Abdullayev, Rickard Sandberg, Manfred Grabherr, Björn Andersson, András Simon

**Affiliations:** 1 Department of Cell and Molecular Biology, Karolinska Institute, Stockholm, SE-171 65 Sweden; 2 College of Animal Science and Technology, Huazhong Agricultural University, Wuhan, 430070 China; 3 Institut Jacques Monod, CNRS & University Paris-Diderot, Paris, 75205 France; 4 Department of Medical Biochemistry and Microbiology, Uppsala University, Uppsala, SE-751 23 Sweden; 5 Department of Stem Cell and Regenerative Biology, Harvard Stem Cell Institute, Harvard University, Cambridge, MA 02138 USA; 6 Ludwig Institute for Cancer Research, Stockholm, SE-171 65 Sweden; 7 Present Address: The Francis Crick Institute, NW1 1AT London, UK

## Abstract

Salamanders exhibit an extraordinary ability among vertebrates to regenerate complex body parts. However, scarce genomic resources have limited our understanding of regeneration in adult salamanders. Here, we present the ~20 Gb genome and transcriptome of the Iberian ribbed newt *Pleurodeles waltl*, a tractable species suitable for laboratory research. We find that embryonic stem cell-specific miRNAs mir-93b and mir-427/430/302, as well as Harbinger DNA transposons carrying the *Myb*-like proto-oncogene have expanded dramatically in the *Pleurodeles* *waltl* genome and are co-expressed during limb regeneration. Moreover, we find that a family of salamander methyltransferases is expressed specifically in adult appendages. Using CRISPR/Cas9 technology to perturb transcription factors, we demonstrate that, unlike the axolotl, *Pax3* is present and necessary for development and that contrary to mammals, muscle regeneration is normal without functional *Pax7* gene. Our data provide a foundation for comparative genomic studies that generate models for the uneven distribution of regenerative capacities among vertebrates.

## Abstract (web summary)

The Iberian ribbed newt *Pleurodeles waltl* has a wide spectrum of regeneration abilities. Here, Elewa *et al*. sequence its ~20 Gb genome and transcriptome to investigate the molecular features underlying its regenerative capacities.

## Introduction

**Fig. 1:**
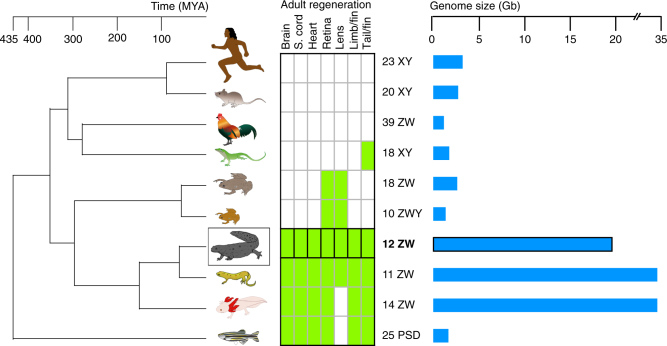
The Iberian ribbed newt *P*. *waltl* is a prime model for adult regeneration. Compared to other research animal models, *P*. *waltl* is the most regenerative adult vertebrate amenable to laboratory breeding. Phylogenetic tree from TimeTree^66^. Regeneration capacities based on^6, 13, 67–70^. Sex chromosomes from refs. ^71–74^. Genome sizes from https://www.ncbi.nlm.nih.gov/genome/ (summed from Size (Mb) column)

The random manifestation of extensive regeneration capacities in the animal kingdom implies a phylogenetically widespread regeneration potential, which is masked in most species^1–5^. Among tetrapods, salamanders, such as newts and axolotls, display the largest regenerative repertoire. A newt can rebuild entire limbs, tails, jaws, cardiac muscle, ocular tissues, and restore central nervous system tissues including brain structures^6^ (Fig. 1). However, it is important to note that major differences exist even among salamanders. In contrast to the paedomorphic axolotl, newts undergo metamorphosis, have a broader regeneration spectrum and mobilize additional cell sources for regeneration of the same body part^7^. Such interspecies differences among closely related species offer opportunities to reveal valuable information about the evolution of processes that allow or counteract regeneration. Although significant progress has been made to characterize salamander transcriptomes and proteomes^8–12^, features such as species-specific genes, expansion or contraction of gene families, and the underlying cause of their gigantic genome size remain largely unexplored. In addition, due to their complex and long life cycle, most newt species are cumbersome to breed under laboratory conditions, which has hampered the establishment of genetically modified lines. However, the Iberian ribbed newt *Pleurodeles waltl* (*P*. *waltl)* is easily bred in laboratories and retains the widest known spectrum of regeneration abilities among adult vertebrates^13^ (Fig. 1; Supplementary Fig. 1a). Here we describe the genome and transcriptome of *P*. *waltl* (Methods; Supplementary Methods, Supplementary Fig. 1b, and Supplementary Tables 1–5) as a resource to explore regeneration relevant novelties, and adapt CRISPR/Cas9 technology to perturb key transcription factors involved in regeneration.

## Results

### Sequencing the genome and transcriptome of *P*. *waltl*

The diploid genome of *P*. *waltl* is organized in 12 chromosome pairs, which have been the subject of classical lampbrush chromosome studies^14^ (Supplementary Fig. 1c–h). The *P*. *waltl* haplotype genome size is ~20 Gb (Supplementary Table 1), making this one of the largest vertebrate genomes sequenced to date. Our genome annotation pipeline identified 14,805 complete protein-coding gene models and we estimate that this set represents 64.8% of *P*. *waltl* protein-coding genes (Methods; Supplementary Methods, Supplementary Fig. 2a). The remaining gene content was reconstructed in the de novo transcriptome assembly involving RNAseq data from embryonic, larval, different adult tissues, and limb regeneration stages (Methods; Supplementary Methods, Supplementary Tables 3–5). We estimate that this combined set of gene models and transcripts represents 98.1% of the *P*. *waltl* protein-coding repertoire (Supplementary Methods; Supplementary Table 6). To provide a platform for comparative genomic studies, we identified 19,903 orthology groups involving *P*. *waltl* protein-coding genes and/or transcripts (Supplementary Methods; Supplementary Fig. 2a, Supplementary Table 1, Supplementary Data 1). Of these orthology groups, 1575 consisted of salamander members only (salamander groups) and 1130 consisted of salamander and *Xenopus* orthologs only (amphibian groups). The remaining 17,198 groups consisted of salamander and other vertebrate orthologs (human, mouse, chicken, lizard, or zebrafish). Importantly, we did not observe any expansion or loss of non-transposable protein-coding orthologs compared to other vertebrates (more than twofold increase or decrease) (Supplementary Methods, Supplementary Data 1–3).

### An expansion of Harbinger elements in the *P*. *waltl* genome

**Fig. 2:**
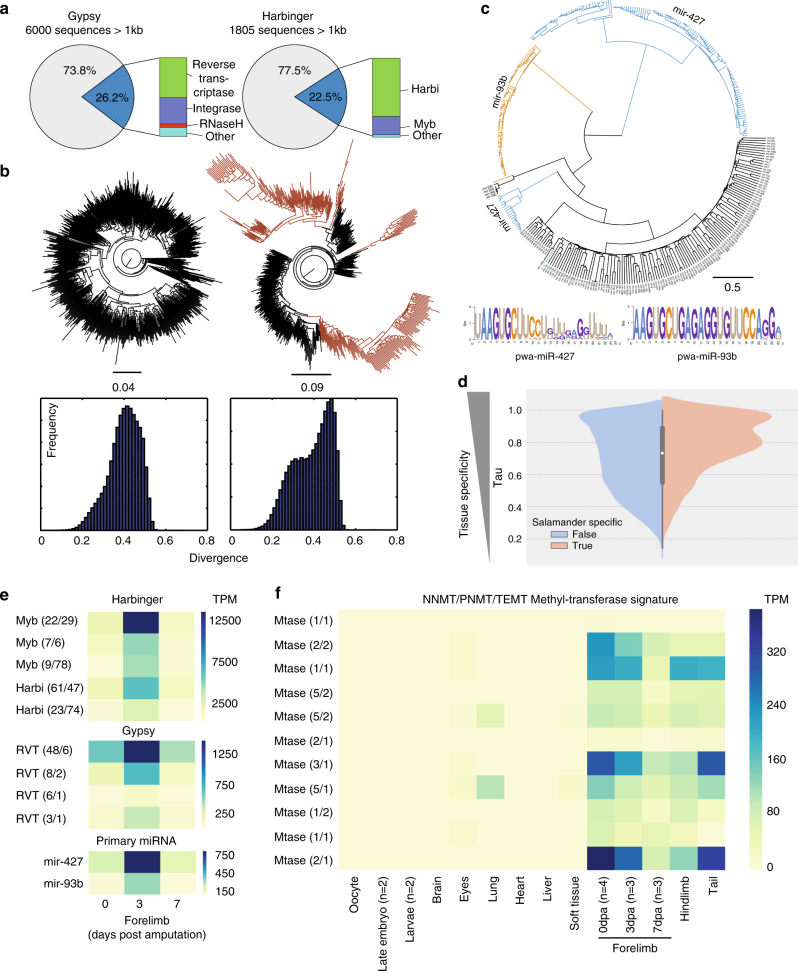
Genomic expansion and the expression of Harbinger DNA transposable elements pluripotency miRNAs and salamander-specific methyltransferases during adult limb regeneration in *P. waltl*. **a** Portion of Gypsy and Harbinger repeats (>1 kb) retaining a detectable protein domain. **b** Phylogeny of Gypsy and Harbinger elements. While the tree-based on Gypsy elements indicates a uniform expansion, the tree-based on Harbinger elements shows three branches (brown) resulting from a second distinct burst. The second Harbinger burst also results in a bimodal pairwise genomic distance distribution, compared to a unimodal distribution for the Gypsy elements. **c** Phylogenic tree of 361 mature *P*. *waltl* miRNAs with miR-427 and miR-93b expansion highlighted (top). Sequence logo based on multiple alignment of 155 miR-427 sequences and 66 miR-93b sequences (bottom). Note the embryonic stem cell-specific cell cycle regulating seed AAGUGC within the first eight nucleotides. **d** Split violin plot showing the tau score distribution of *P*. *waltl* genes that have non-salamander vertebrate orthologs (blue) and genes that only have salamander orthologs (beige). **e** RNAseq quantification of five Harbinger orthology groups encoding Myb or Harbi domain-bearing proteins (top), four Gypsy orthology groups encoding reverse transcriptase domain-bearing proteins (middle) and mir-427 and mir-93b primary transcripts (bottom). In parentheses are the number of transcripts and gene models belonging to each group. The groups chosen were significantly upregulated in the regenerating limb 3 days post amputation (dpa). Differential expression calculated by DESeq2 (Supplementary Table 9). TPM transcripts per million. **f** Eleven orthology groups of salamander methyltransferase are expressed specifically in appendages of the adult animal. In parentheses are the number of transcripts and gene models belonging to each group

A striking feature of the *P*. *waltl* genome is the extent and diversity of its repetitive elements. The genome is host to a diverse population of class I and class II transposable elements (Supplementary Table 1). We assembled a repeat library by majority vote k-mer extension^15^, followed by alignment to the repeat database RepBase^16^ using Satsuma^17^, yielding 428 distinct sequences (Methods). Gypsy retrotransposons are the most frequent repetitive elements in *P*. *waltl*, followed by the Harbinger transposons, and together account for about two thirds of the genome repetitive content (Supplementary Table 1). A phylogeny of ~1200 Gypsy elements longer than one kilobase indicates continuous expansion of this family (Fig. 2a, b), while Harbinger elements have undergone two distinct evolutionary bursts, with one recent expansion, visible from the distribution of pairwise similarity (Fig. 2a, b; Supplementary Fig. 2b). Harbinger elements are distinct from other transposons in that they carry a *Myb*-like gene, a proto-oncogene that acts as a transcription factor. While Harbinger elements gave rise to the genes *Harbi1* ^18^ and *Naif1* ^19^ in the vertebrate lineage, their contribution to vertebrate genome content is extremely rare with the leading example being the genome of coelacanth *Latimeria chalumnae* (~1 to 4% of the genome)^20^. Therefore, the Harbinger element expansion we describe in *P*. *waltl* is hitherto unprecedented.

### Expansion of embryonic stem cell-specific miRNAs in *P*. *waltl*

MicroRNAs (miRNAs) are short non-coding RNAs that regulate post-transcriptional gene expression^21–23^. A number of miRNAs, including miR-302, are capable of reprogramming mammalian somatic cells into an induced pluripotent state^24^. We identified 361 miRNA precursors in the *P*. *waltl* genome that produce 67 distinct mature miRNAs conserved in *Xenopus*. In addition, the de novo transcriptome included 202 transcripts bearing conserved miRNA precursors (primary miRNA transcripts) that produce 55 of the 67 genome predicted mature miRNAs (Supplementary Methods, Supplementary Fig. 3, Supplementary Tables 7–8, Supplementary Data 4). Surprisingly, most of the genome predicted precursors were copies of mir-93b^25^ and mir-427^26^ (known as mir-430 in zebrafish^27^ and mir-302 in mammals^28^) (66 and 155 precursors, respectively) (Fig. 2c). Both mir-93b and mir-427 bear the characteristic embryonic stem cell-specific cell cycle regulating (ESCC) seed (AAGUGC) and are of interest to regeneration studies^29^ (Fig. 2c). The expansion of mir-427 has been reported in *Xenopus* (~80 copies)^25^ and zebrafish (~100 copies)^30^ and thus predates the Devonian period^31^. The mir-93b expansion, however, appears to be salamander-specific and we place its occurrence in the Jurassic period or later^32^.

### Harbinger and ESCC miRNA expression during limb regeneration

Given the compelling expansion of ESCC miRNAs and Harbinger transposable elements, we analyzed whether mir-93b, mir-427, Myb, and Harbi domain containing genes are regulated during adult *P*. *waltl* limb regeneration (Supplementary Table 9). We mapped total RNAseq reads from three early developmental stages, nine adult (>2 years old) body parts and two limb regeneration stages; 3 and 7 days post amputation (Supplementary Table 3) to 14,805 gene models and 108,713 transcripts belonging to the *P*. *waltl* orthology groups in addition to 202 miRNA primary transcripts (Supplementary Methods, Supplementary Data 1,4). We found that mir-93b and mir-427 primary transcripts are upregulated in the regenerating limb 3 days post amputation (*p* < 0.001) and that their mature miRNAs are detected during limb regeneration (21 and 45 reads per million, respectively) (Fig. 2e; Supplementary Fig. 4, Supplementary Table 9 and Supplementary Data 4). In addition, we found that five orthology groups containing Myb or Harbi domains were upregulated at 3 days post amputation (*p* < 0.001) (Supplementary Table 9) similar to mir-93b and mir-427 primary transcripts (Fig. 2e). On the contrary, the two vertebrate Harbinger derivative genes *Naif1* and *Harbi1* were expressed at low levels in all our datasets and showed no regulation during regeneration (Supplementary Table 10). Four Gypsy orthology groups were also upregulated 3 days post amputation (*p* < 0.01), however, their expression levels were an order of magnitude lower than the Harbingers (Supplementary Table 9). Transposable elements are often domesticated to benefit their hosts and RNA transposable element expression has been described in mammalian pluripotent stem cells^33, 34^. Our data illustrate that the both RNA and DNA transposons, Gypsy and Harbinger, respectively, in addition to ESCC miRNAs respond to adult salamander injury. The extent to which they co-regulate regeneration awaits further studies.

### Restricted expression of salamander methyltransferases

Genes found only in salamanders may offer insight into their unique regenerative abilities. Our annotation pipeline identified 1545 orthology groups that consisted of genes and transcripts in the *P*. *waltl* genome and at least one other salamander, but no sequences from the other seven model vertebrates analyzed. We consider these orthology groups to be putatively salamander-specific and refer to them by the protein domain(s) detected in their members. We calculated the tau score^35^ (an indicator of tissue specificity) for all orthology groups and found that groups with only salamander genes are more tissue specific than groups with orthologs in other vertebrates (Fig. 2d and Methods). Surprisingly, we found eleven salamander orthology groups encoding a NNMT/PNMT/TEMT methyltransferase domain and expressed specifically in adult limbs and tail, four of which were significantly downregulated on the seventh day of regeneration (*p* < 0.001; Fig. 2f; Supplementary Fig. 2b and Supplementary Table 9). No other salamander orthology groups showed this expression pattern. Together, our data show that *P*. *waltl* has evolved genes with tissue-restricted expression profiles and that target methylation may have evolved in a manner consistent with limb regeneration.

### *Pax3* and *Pax7* are present and functional in *P*. *waltl*

**Fig. 3:**
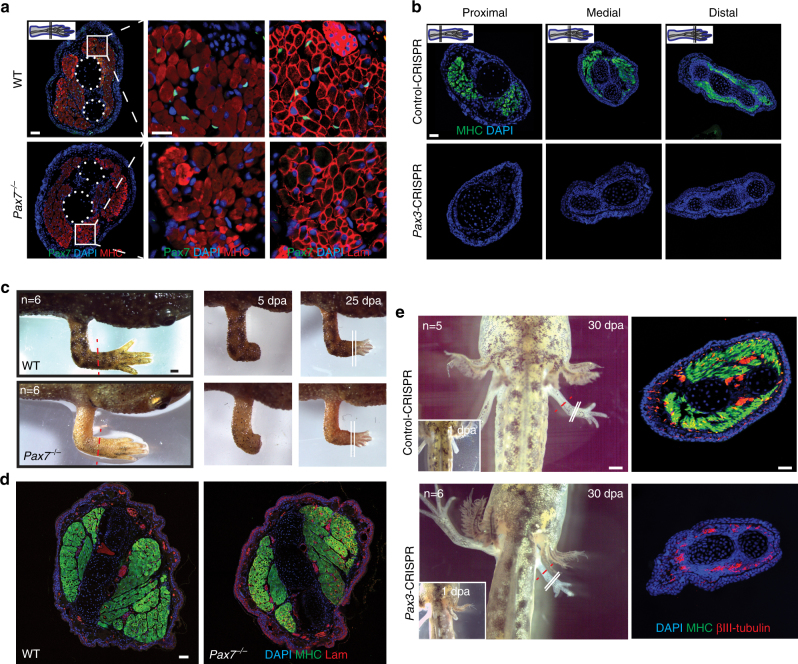
Newt limb muscle development and regeneration is independent of *Pax7*. **a** *Pax7* ^−/−^ F_1_ *P*. *waltl* have normal limb skeletal muscle. Note the similar myosin heavy chain (MHC) and laminin (Lam) staining in *Pax7* ^−/−^ and WT animals. Scale bars = 200 µm in the overview and 20 µm in the insert. **b** *Pax3*-CRISPR mosaic animals have muscle-less limbs. Immunofluorescent staining for MHC in transverse sections of *Pax3*-CRISPR and control-CRISPR limbs. Scale bar = 100 µm. **c** Limb regeneration is not impaired in post-metamorphic *Pax7* ^−/−^ F_1_ animals. Scale bar = 1 mm. **d** *Pax7* ^−/−^ F_1_ animals regenerate normal skeletal muscle in the new limb. Note the indistinguishable myosin heavy chain (MHC) and laminin staining in *Pax7* ^−/−^ and WT animals. Scale bar = 100 µm. **e** muscle-less limbs in *Pax3*-CRISPR mosaic animals (stage 45) regenerate muscle-less limbs. Immunofluorescent staining for MHC and the neuronal marker βIII-tubulin in transverse sections. Scale bar = 1 mm in the left panels showing animals and 100 µm in the right panels showing sections. Red dotted lines indicate amputation plane. White lines indicate section plane

We were intrigued by the absence of *Pax3* in the axolotl (*Ambystoma mexicanum*) transcriptome, genome and previous gene expression studies^10, 36–38^. *Pax3* is a paired-end homeodomain transcription factor required for early development^39, 40^ and its paralog *Pax7* regulates skeletal muscle regeneration^7, 41^. Manual curation of the *Pax* gene family and in situ hybridization confirmed the presence of *Pax3* and *Pax7* in *P*. *waltl* (Supplementary Fig. 5a–c). We adapted CRISPR/Cas9 technology to *P*. *waltl* and mutated both genes (Methods; Supplementary Fig. 5a,d and Supplementary Table 11). Whereas *Pax7* ^−/−^ compound heterozygote F_1_ animals exhibited no apparent deficiencies in muscle development, *Pax3* mosaic-mutants died or developed animals with several developmental anomalies, exemplified by muscle-less limbs (Fig. 3a, b; Supplementary Fig. 5e–f and Supplementary Video 1). Next we asked how these mutations affect limb regeneration. *Pax7* ^−/−^ F_1_ animals regenerated limbs indistinguishable from wildtype controls, including skeletal muscle and cells in satellite cell position (Fig. 3c, d and Supplementary Fig. 6; Methods). *Pax3* mosaic F_0_ mutants lacking limb skeletal muscle regenerated morphologically normal muscle-less limbs without any other overt phenotypes (Fig. 3e).

## Discussion

Salamander genomes are gigantic, ranging in size between 14 and 120 Gb^42^. While this feature has facilitated genome structure studies using lampbrush chromosomes^43^, it has delayed sequencing projects and impeded regeneration studies. Lampbrush chromosomes epitomize genome structural plasticity, while salamander regeneration is a prime example of cellular plasticity. Whether these two forms of plasticity are mechanistically related is now a pursuable question.

Reading and editing the *P*. *waltl* genome reveals several new features of limb regeneration and genome evolution. Our loss of function data on *Pax3* and *Pax7* paralogues show that limb regeneration is not dependent on skeletal muscle and that no other tissues give rise to muscle during limb regeneration in *P*. *waltl* ^44^. Furthermore, the expression and developmental functions of *Pax3* are conserved between *P*. *waltl* and mammals^41^, which also raises the question whether the lack of *Pax3* in the axolotl confers additional functions for *Pax7*. Mammalian skeletal muscle regeneration is impaired in the absence of *Pax7* ^45^ but we have found that *Pax7* loss of function does not impair muscle regeneration in *P*. *waltl*. In this context, it is important to note that a distinctive process during newt limb regeneration is the reversal of terminal differentiation of skeletal muscle fibers. Myogenic dedifferentiation during limb regeneration produces progenitor cells that build up the new muscle and thus has the potential to compensate for the potential loss of contribution from satellite cells^7^. However cell tracking studies of satellite cells and their progeny are necessary to resolve this question^46^.

The genomic expansion and expression of ESCC miRNAs and Myb domain-bearing Harbingers, along with limb-specific expression of putatively salamander-specific methyltransferases present new possibilities to uncover mechanisms of adult vertebrate regeneration using *P*. *waltl* as a model organism. For example, the multitude of mir-427 copies in fish and amphibian genomes in addition to that of mir-93b in newts may provide an opportunity for individual copies to evolve and acquire expression rights and co-regulate a regenerative response after injury. In *X*. *laevis* and zebrafish, miR-427/430 helps clear of cache of maternally supplied RNA when embryos transition to rely on their own gene expression^47, 48^. One intriguing possibility is that miR-427 performs a similar function in *P*. *waltl* during limb regeneration. In this model, miR-427 clears the cytoplasmic cache of mRNAs, thereby erasing a cell’s “working memory” and allowing dedifferentiation to occur, while the “deep memory” of the cell’s identity and function would not be lost by a permanent inactivation of gene expression.

## Methods

### Genomic DNA isolation

High molecular weight genomic DNA was purified from a single larva after discarding the digestive system. The larva represents the first generation of our *P*.*waltl* colony, established from fertilized eggs produced in a laboratory colony located in Madrid, Spain. The larva is therefore the fourth/fifth generation of laboratory-bred *P*. *waltl*, originally obtained with permission from a wild population in Doñana National Park (Spain) for research purposes by Agustin Gonzalez. The body was segmented into five pieces and each piece was placed in 1.5 mL centrifuge tube. A total of 700 μL digestion buffer (50 mM Tris-HCl pH 8.0, 100 mM EDTA, 100 mM NaCl, 1% SDS) was added to each sample. 50 μL 10 mg mL^−1^ Proteinase K was added to the sample and incubated 55–60 °C overnight with mixing. Another 50 μL of 10 mg mL^−1^ Proteinase K was added in the morning and the samples incubated for 4 more hours. Next, 20 μL 10 mg mL^−1^ RNase A (DNase-free) was added to the sample and incubated at 37 °C for 1–2 h. The solution was transferred to a pre-spun (1500 × *g* for 1–2 min) phase lock gel (PLG) 2 mL Heavy tube. 0.5 mL phenol-chloroform-isoamyl alcohol (25:24:1) was added to the sample in the PLG 2 mL tube and mixed well by repeated inversion. Next, tubes were centrifuged at full speed for 5 min in a microcentrifuge. The resultant aqueous phase was transferred to a fresh pre-spun PLG 2 mL Heavy tube. A total of 0.5 mL chloroform-isoamyl alcohol (CI, 24:1) was added to the sample in the PLG 2 mL tube and mixed well by repeated inversion. Tubes were centrifuged at full speed for 5 min in a microcentrifuge. The resultant aqueous phase was transferred to a fresh microcentrifuge tube. 100% isopropanol was added and mixed thoroughly by repeated inversion. High molecular weight DNA was recovered using a heat-sealed glass micropipette tip and transferred to a 1.5 mL microcentrifuge tube containing 70% ethanol. The glass tip with DNA was then dipped into 95% ethanol twice. DNA was allowed to dry partially before being pushed off the tip into a microcentrifuge tube containing 400 μL TE and resolubilized overnight. DNA quality was assessed using DropSense and gel electrophoresis.

### Karyotyping

Cells were arrested in metaphase using classical procedures, standardized for *P*. *waltl* ^49^: stage 20 embryos (*n* = 6) were placed in saline solution (30 mM NaCl, 0.34 mM KCl, 0.9 mM CaCl_2_) containing 0.5% colchicine. After 18 h at 21 °C, the embryos were fixed with a mix of 50% acetic acid/ 50% EtOH for 3–5 min, washed in distilled water and transferred to 50% acetic acid, were they were dissociated mechanically with a Pasteur glass pipette. Cells were then dropped on Superfrost slides, let dry, and stained with DAPI for confocal analysis. For Supplementary Figure 1, a Z-stack confocal projection was made with an interval of 0.57 μm between planes, and the twelve pairs of chromosomes were identified and distributed in three groups according to ref. ^50^. For an easy interpretation of the reader, colors were given applying color filters to individually selected chromosomes using Adobe Photoshop. The full extent of the chromosomes in the Z-projection was then selected, filled with color, and organized to provide the karyotype.

### Lampbrush chromosome analyses

Ovarian biopsies were performed on adult females of *P*. *waltl* that were anesthetized in 0.15% MS222 (Amino-benzoic Acid Ethyl, Fluka). Stage IV–VI oocytes were selected and maintained in MBS buffer (Modified Barth’s solution) at 18 °C. Germinal vesicles were manually isolated from the oocytes and dissected in 75 mmol L^−1^ KCl, 25 mmol L^−1^ NaCl, 0.01 mmol L^−1^ MgCl2 and 0.01 mmol L^−1^ CaCl_2_, pH7.2, and LBCs were prepared as previously described^51^. Nuclear spread preparations were centrifuged at 300 × *g* for 10 min, and at 3000 × *g* for 30 min at 4 °C. Standard transmitted light were performed at the ImagoSeine core facility (member of the IBiSA and the France-BioImaging (ANR-10-INBS-04) infrastructure) using a wide field Leica microscope with phase contrast plan Apo oil objectives: ×40 (NA = 1.3), ×63, ×100 (NA = 1.4). Images were captured using a CoolSnap HQ, Photometrics camera driven by the software Metamorph6 (Universal imaging). Series of 11 confocal Z-planes (0.7 µm distance) were collected. Pixel size of the images were 0.1625 μm, 0.103 μm, and 0.065 μm for the ×40, ×63, and ×100 objectives, respectively.

### Genome size calculation

Nuclear content was measured using *Vicia faba* nuclei as reference (2C DNA content = 26.9 pg)^52^ (Supplementary Fig. 1). All samples were prepared according to^52^ and stained with 4ʹ,6-diamidino-2-phenylindole (DAPI) and measured using a BD Influx™ by Becton Dickinson flow cytometer. The lasers used for detection and excitation were 488 nm and 355 nm, respectively. From the mean peak positions of *P*. *waltl* (20,255) and *V*. *faba* (13,746) the 2 C DNA content was calculated as 39.6 pg using the equation:

*P*.*waltl* 2 C value = *V*.*faba* 2 C value (reference) × ((*P*.*waltl* 2 C mean peak position)/(*V.faba* 2 C mean peak position))

Based on the equation:

haploid genome size = (0.978 × 2 C value)/2, the haploid *P*. *waltl* genome size was calculated to be 19.38 Gb (Supplementary Fig. 1).

Our estimates agree with Licht and Lowcock (2 C value = 42.7 pg)^53^, Morescalchi and Olmo (2 C value = 39 pg)^54^ and are lower than Lizana and collegues (2 C value = 48.25 pg)^55^.

### Short read genome sequencing

Libraries for Illumina sequencing were generated and sequenced at SciLifeLabs (Stockholm). Two Illumina TruSeq PCR-free libraries (insert size 180 bp) were prepared and paired-end 2 × 125 bp sequencing was done on HiSeq2500 (HiSeq Control Software 2.2.58/RTA 1.18.64) with a setup in High Output mode (Supplementary Table 2).

### Genome assembly strategy

Illumina paired-end reads from two 180 bp insert size genomic libraries were assembled into contigs using Abyss (28) using a *k* = 71. Illumina paired-end RNAseq reads from representative body sections of *P*. *waltl* were assembled into transcripts using Trans-Abyss (29) (*k* = 69) with the aim to reconstruct only longer, complete transcripts rather than isoforms or short RNAs. Trans-Abyss transcripts were then used to scaffold the genomic Abyss-contigs in a third assembly aimed at improving the reconstruction of gene content. The final assembly—denominated Pw_v4.2—was 19.66 Gb in size and included ~66 million contigs and 106,895 scaffolds (Table 1). Approximately 45% of the genome assembly was captured in contigs ranging between 1 kb and 10 kb in size and ~6% (1.2 Gb) were assembled in contigs and scaffolds longer than 10 kb, the longest being 438.8 kb in size (Supplementary Fig. 1b).

### Repeat library assembly

We assembled a repeat library by majority vote k-mer (*k* = 25) extension algorithm^15^ to the *P*. *waltl* genome assembly Pw_v4.2, resulting in a set of 428 consensus repeat sequences larger than 1000 nt. Out of these, 349 were identified as known repeats through nucleotide alignments against RepBase (version 21.10 downloaded 10/23/2016)^16^ using Satsuma (https://sourceforge.net/projects/satsuma/ last update 2016-12-14)^17^ and selecting the longest alignments respectively if multiple overlapping hits were reported.

### Phylogeny of Gypsy and Harbinger elements

We computed pairwise genomic distances using Satsuma for 6000 randomly selected Gypsy and Harbinger elements longer than 1000 nucleotides, requiring alignments to cover the same region in each element. This resulted in 625 Gypsy and 567 Harbinger elements, for which we built phylogeny using the neighbor joining method implemented in phylip^56^, and rooting the tree on midpoint. For bootstrapping, the longest 44 and 50 sequences for Gyspy and Harbinger elements, respectively, were aligned using MUSCLE^57^, from which Maximum Likelihood trees were built using PhyML^58, 59^. Bootstrap values were estimates from 100 trials.

### Transcriptome RNA preparation and sequencing

25 indexed Illumina TruSeq Stranded total RNA Zero-Gold libraries (mean insert size 150 bp) from 20 *P*. *waltl* body parts and regeneration stages were combined into four pools and sequenced on 4 lanes (one pool per lane) on Illumina HiSeq High Output mode v4, PE 2 × 125 bp at the Science for Life Laboratory, Stockholm (Supplementary Table 3). In all cases, RNA was isolated using Trizol after grinding the sample with a mortar and pestle in liquid nitrogen and precipitated overnight in isopropanol. RNA integrity was assessed using Agilent Bioanalyzer. Samples with RIN ≥ 8.0 were used for library preparation.

### Genome annotation generation of gene models with MAKER

MAKER was used in a two-step approach to identify gene models in the *P*. *waltl* genome.

MAKER Tier 1: The clustered assembled transcripts, the Augustus hinted gene models and the protein sequences from uniref90 were used as input evidence to build the annotation with MAKER.

MAKER Tier 2: Nucleotide gene models from the MAKER Tier 1 annotation were used as input for the second round of gene annotation, instead of the Augustus hinted gene models.

In total, 79,916 gene models were identified using this approach. 19,915 gene models aligned with a transcript in the de novo assembled transcriptome (percent identity > 98%, alignment length > 99 bp).

### Genome editing

Cas9 mRNA and gRNA were synthesized using mMESSAGE T7 ULTRA Transcription Kit (Invitrogen) and MEGAshortscript T7 Transcription Kit (Invitrogen), respectively. Genomic DNA was extracted with Qiagen DNAeasy kit. The PCR products were cloned into TOPO cloning vector (Invitrogen). Individual clones were sequenced with T7 primer (Supplementary Table 9).

Single-cell fertilized eggs were obtained by either natural or induced breeding and injected according to previously published protocols with modifications^60, 61^. Briefly, 500 pg Cas9 RNA and 100 pg gRNA were mixed into 5 nl and injected into freshly laid single-cell-stage embryos. The animals were raised according to^60^. The screening of F_0_ animals was done by both genotyping (see above) and phenotype characterization, including pigmentation analysis and immunohistochemistry of limbs/tails, according to^39, 40^. The *Pax7* ^−/−^ and *Tyr* ^−/−^ F1 animals were produced by crossing between adult F_0_ animals. The larvae or post-metamorphic newts were anaesthetized within 0.01% or 0.1% ethyl-p-aminobenzoate (benzocaine; Sigma) prior to imaging and tissue collection. Newts utilized for this study were processed according to Swedish Board of Agriculture animal ethical regulations (N429/12).

### In situ hybridization and immunohistochemistry

The PCR fragments of *Pax3* and *Pax7* were cloned into TOPO cloning vectors. The digoxigenin-labeled antisense RNA probes were synthesized using T7 RNA polymerase (Roche). Whole-mount in situ hybridizations were performed by using albino Tyr ^−/−^ F1 embryos according to^62^. In situ hybridizations on 10 µm transverse sections were carried out by fixing sections in 4% formaldehyde for 15 min, treating with 0.2 N HCL for 12 min, washing, and then incubating in acetylation buffer (0.1 M trietholamine and 0.25% acetic anhydride) for 10 min. Next, the slides were rinsed in RNase free water and permeabilized with a solution of 1 μg mL^−1^ Proteinase K (Roche) and 2 mM CaCl2 for 15 min at 37 °C. The slides were incubated in prehybridization buffer (50% deionized form of amide, 5 × saline sodium citrate (SSC), 5 × Denhardt’s solution, 250 μg mL^−1^ yeast RNA, 500 μg mL^−1^ herring sperm single-stranded DNA (ssDNA); Sigma) for 2 h at room temperature before being incubated in hybridization solution (50 ng probe, 50% deionized form of amide, 5 × SSC, 5 × Denhardt’s solution, 250 μg mL^−1^ yeast RNA, 500 μg mL^−1^ herring sperm ssDNA) for 16 h at 60 °C. The slides were then washed with 0.2 × SSC buffer containing 0.05% tween 20 at 70 °C for 3 × 1^63^. Finally, BM purple was used to visualize the signal. For immunostainings, sections were blocked with 10% donkey serum in 0.1% Triton-X for 30 min at room temperature. Sections were incubated with a relevant primary antibody overnight at 4 C and with secondary antibodies for 1 h at room temperature. Antibodies were diluted in blocking buffer and sections were mounted in mounting medium (DakoCytomation) containing 5 mg mL^−1^ 1 4,6-diamidino-2-phenylindole (Sigma)^64^.

### Cryosectioning and in situ hybridization for miRNAs

Limb samples were collected at the indicated timepoints and fixed overnight at 4 °C, in freshly made 4% PFA in PBS. After fixation, samples were washed three times in PBS and PBST, equilibrated in 30% sucrose in PBS, embedded in Tissue Tek (Sakura, Torrance, CA, USA) and frozen for cryosectioning. Frozen tissues were sectioned into 20 μm-thick longitudinal sections.

Tissue sections were dehydrated through a MeOH/PBST series (25, 50, and 75%), followed by MeOH for 10 min and rehydrated through the opposite series of MeOH. The sections were then permeabilized with 2 μg mL^−1^ proteinase K (Thermo Scientific), inactivated with glycine solution (2 mg mL^−1^ in PBST) and washed in PBST. Before hybridization, the slides were incubated with hybridization buffer (50% formamide, 5× SSC, 50 µg mL^−1^ heparin, 0.2% Tween-20, and 100 μg mL^−1^ yeast tRNA (Sigma)) for 1 h at RT. Digoxygenin-labeled locked nucleic acid (LNA) probes against the mature form of miRs-93b and 427 were custom-made and ordered from Exiqon (Denmark). The probes were diluted in hybridization buffer (50 nM) and denatured for 5 min at 95 °C. Hybridization was carried overnight at 53 °C. The next day proceeded with washes in 5× SSC for 10 min and 0.2× SSC for 1 h at hybridization temperature. Then, slides were incubated in blocking buffer (10% sheep serum and 2% bovine serum albumin in PBST) at RT for 30 mins. Alkaline phosphatase associated anti-digoxigenin antibody (Roche) was diluted 1:1000 in blocking buffer, and slides were kept in the antibody solution at RT for 90 min. After washing the antibody with PBST thrice, sections were incubated with freshly prepared alkaline phosphatase (AP) buffer (100 mM Tris-HCl, pH 9.5, 50 mM MgCl2, 100 mM NaCl, 0.2% Tween-20 in water) three times for 5 min. Finally, BM Purple (Roche) was used to stain the sections, in the dark, until development of a blue/purple color. Slides were rinsed with PBST several times, and a drop of 0.5 M EDTA was added to stop the reaction. Slides were mounted with 80% glycerol and imaged using a Olympus Microscope (Center Valley, PA, USA) at ×10 magnification.

Probe Sequences:

miR-93b: 5ʹ-TCATGGAACACCTCTCAGCACTT-3ʹ

miR-427: 5ʹ-AACCTCAACAGGAAGCACTTA-3ʹ

scramble: 5ʹ-GTGTAACACGTCTATACGCCCA-3ʹ

### Small RNA sequencing

Libraries from small RNAs were prepared using TruSeq Small RNA preparation kit. A total of 1 μg of total RNA from 10 samples were processed according to manufacturer instructions. Libraries were sequenced on Illumina Miseq (Rapid mode), single read 1 × 50 bp.

### microRNA annotation

miRNAs were named based on the *Xenopus* miRNA sharing the same seed.

### microRNA quantification

miRNA expression levels were quantified using miRDeep2 (quantifier.pl). To identify miRNAs associated with regeneration. Since our small-RNA dataset was intended for discovery, not differential expression analysis we did not have replicates for each source. To mitigate the lack of replicates in pursuit of miRNAs associated with regeneration, we divided the miRNA expression data into three groups: adult tissue (brain, eyes, heart, liver, lung), regenerating limb (limb 3 dpa, limb 7 dpa); and embryo (larvae, late embryo). Then we tested whether there was significant miRNA up or downregulation between these groups based on a Gauss–Laplace distribution, as implemented in moose2^65^ (http://grabherr.github.io/moose2/).

The top 10 miRNAs upregulated in the regenerating group compared to the adult group (ranked by Bonferroni-corrected *e*-value) were also the top 10 upregulated in the embryonic group compared to the adult group.

### Gene expression tissue specificity

We used the tau score to quantify tissue specificity of salamander-specific orthology groups compared to orthology groups with members from other vertebrates. A tau score represents the average ratio of a gene’s expression between each tissue and the tissue with the highest expression of that gene. Note that “tissue” often refers to a body part that is a composite of tissues (i.e., heart, brain, etc). A gene with equal expression across tissues will yield an average ratio of 1. In order to make the tau score intuitive (a higher score corresponds to higher tissue specificity), the average ratio is subtracted from 1. Therefore, a gene with equal expression across tissues will have a tau score of 1–1 = 0. Conversely, if a gene is expressed in one tissue and not expressed in the others, the average ratio will approach zero and after subtracting from 1, the tau score will approach 1. Therefore, a gene is more tissue specific the closer its tau score is to 1.

### Data availability

Datasets are deposited under BioProject PRJNA353981 and BioSample accession SAMN07571895, Runs SRR6001098-SRR6001140. Transcriptome and genome assemblies are available upon request.

## Electronic supplementary material

Supplementary Information

Description of Additional Supplementary Files

Supplementary Movie 1

Supplementary Data 1

Supplementary Data 2

Supplementary Data 3

Supplementary Data 4
