# Supplementary material for: Reading and editing the Pleurodeles waltl genome reveals novel features of tetrapod regeneration
Source: Nat Commun. 2017 Dec 22;8:2286. doi: 10.1038/s41467-017-01964-9 (PMC5741667; doi:10.1038/s41467-017-01964-9)
Supplement: Supplementary file 1 — Supplementary Information [file 41467_2017_1964_MOESM1_ESM.pdf]

Supplementary Notes:

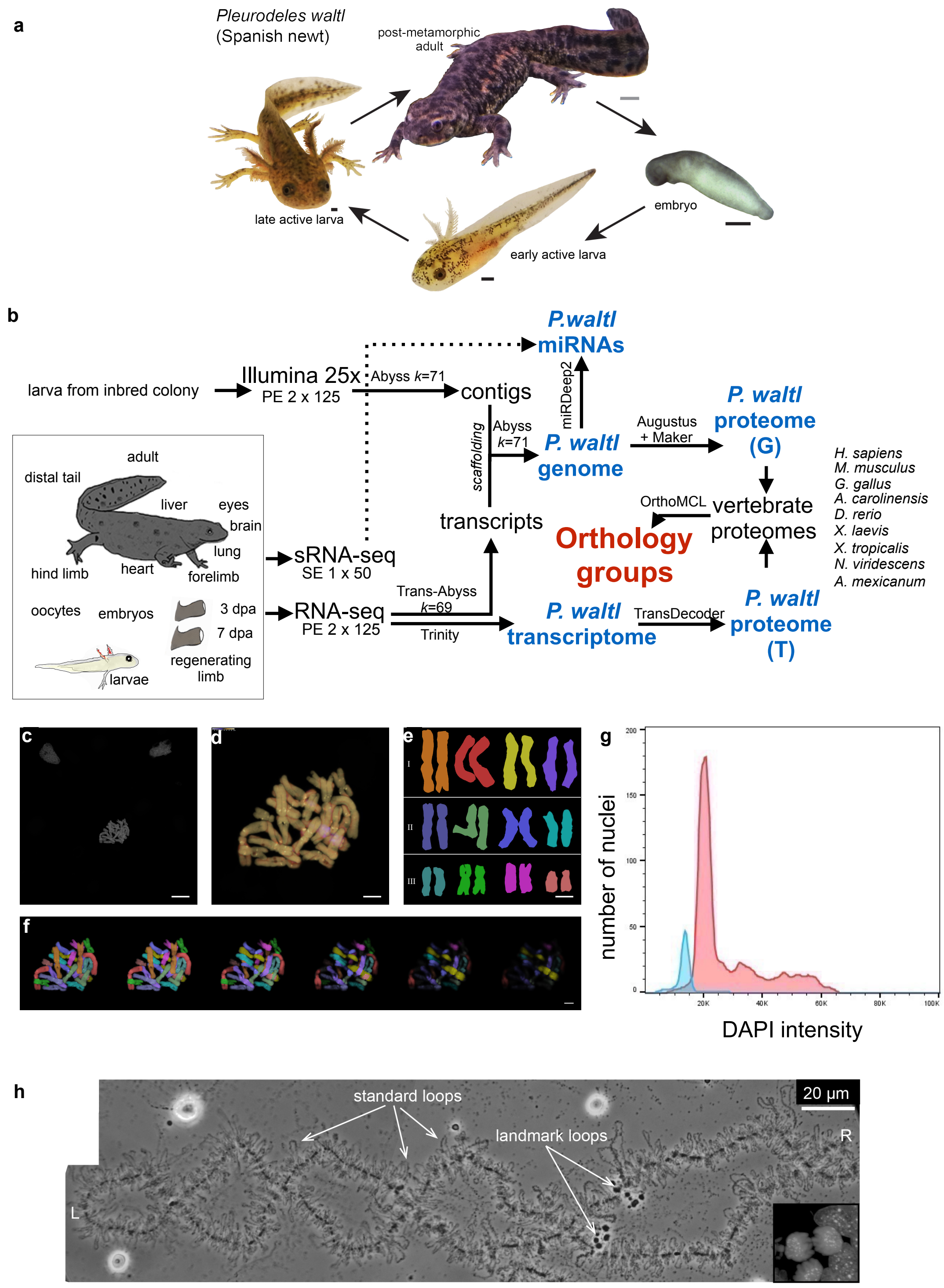

Supplementary Figure 1 | Sequencing strategy for *P. waltl*. a, *P. waltl* life cycle. The generation time under laboratory conditions is 9 – 12 months. Scale bars = 1 mm (black) or 1cm

(grey). **b,** Genomic DNA was sequenced from a single larva after removing the digestive tract. Total RNA and small RNA was harvested from 11 body parts and 2 regeneration stages (Supplementary Tables 2,3). Genome assembly using Abyss was integrated with assembled transcripts to produce a genome assembly, which was the basis of gene model prediction. A reference transcriptome was assembled using Trinity and open reading frames were predicted using TransDecoder. Predicted proteins from the genome gene models and transcriptome transcripts were combined with the proteomes of 9 other organisms to identify orthology groups using orthoMCL. **c,** The 19.38 Gb *P. waltl* genome is organized in 12 chromosome pairs. *P. waltl* cells stained with DAPI after treatment with colchicine: two nuclei in interphase (top) and one cell in the M-phase (center). **d,** Color-coded projection of a Z-stack from the cell in M-phase shown in c. **e,** Karyotype: twelve pairs of chromosomes are organized in three groups in the genus *Pleurodeles*[1]. **f,** Arbitrary color-code was used to recognize the chromosomes in the Z-stack. Scale bars: 20µm (A); 5µm (d-f). **g,** *P. waltl* nuclear DNA quantification. Histograms of relative nuclear DNA contents (relative fluorescence intensities) obtained after the analysis of isolated *Vicia faba* (faba beans; chosen for its large genome; blue) and *P. waltl* (red) nuclei. **h,** The extent of *P. waltl* lampbrush chromosomes enlargement depicted by comparing LBC VI (from diplotene stage oocytes) to chromosomes of a dividing *P. waltl* larval brain cell in anaphase (insert bottom right) at the same magnification. LBC VI is visualized using phase contrast microscopy and shows > 100 standard and 2 landmark loops. (L= left, R = right arm).

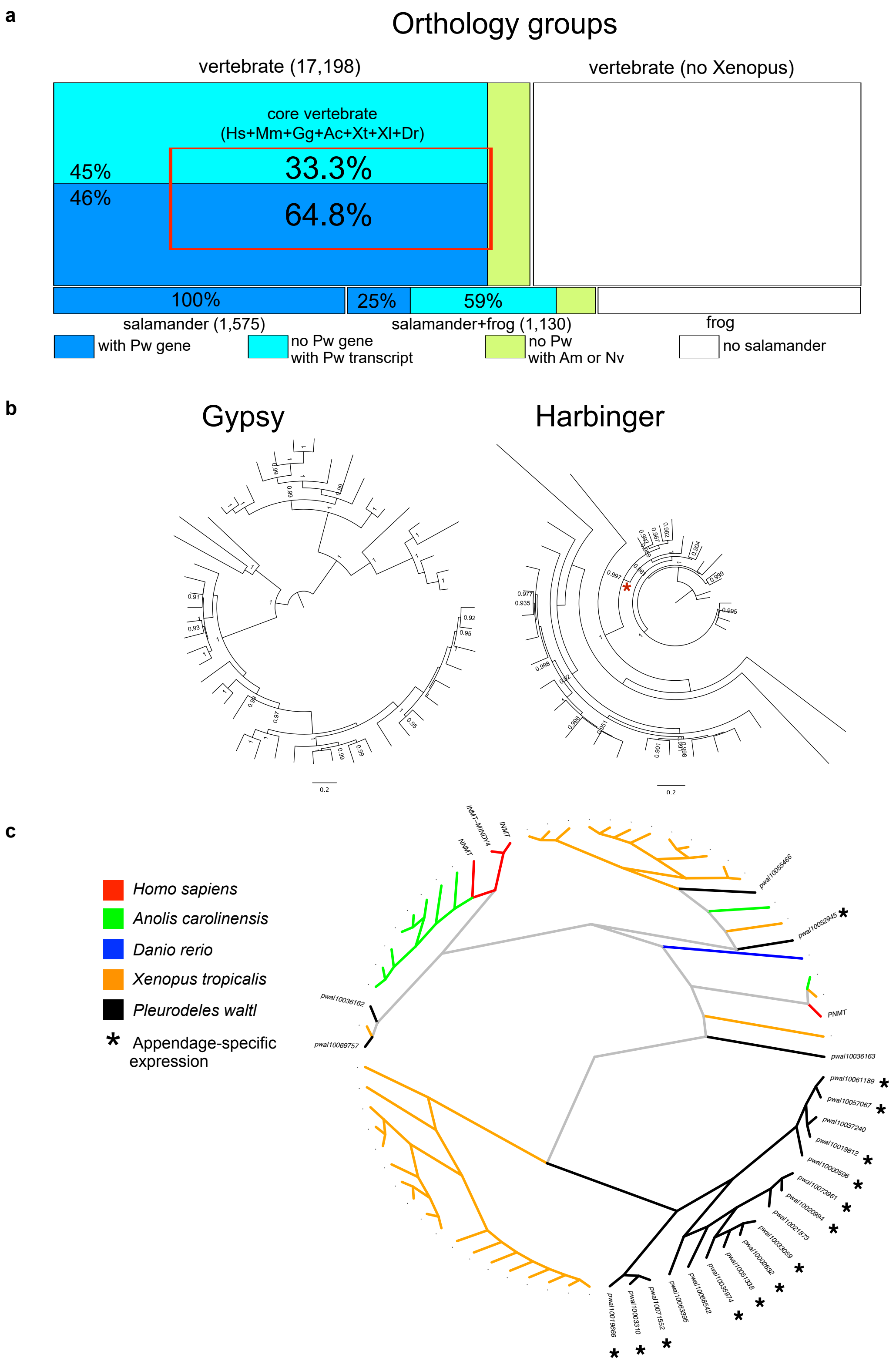

Supplementary Figure 2 |*P waltl* genome and transcriptome analyses reveal Harbinger transposable elements expansion and salamander specific methyltransferases. a, 19,903 *P. waltl* orthology groups (numbers in parentheses). Proportionate areas reflecting the contribution

of each class (vertebrate/amphibian/salamander) to the groups. Blue areas are groups that include at least one *P. waltl* gene model. Cyan groups include at least one *P. waltl* transcript but no gene models. Green groups include at least one *N. viridescens* (*Nv*) or *A. mexicanum* (*Am*) (two salamander species) transcript but no *P. waltl* members. White areas reflect groups devoid of salamander orthologs. Red border delimits 8,259 core vertebrate orthology groups (i.e. include members from all seven non-salamander vertebrate proteomes utilized) from which we estimate that our genome assembly reconstructed 64.8% of *P. waltl* protein-coding genes. **b,** Maximum Likelihood trees for the longest Gypsy and Harbinger elements. Bootstrap values are shown if larger than 0.9. Both trees resemble the larger trees shown in Fig. 2b. The split in the Harbinger tree marked by an asterisk supports the recent expansion highlighted in brown in Fig. 2b. **c,** Methyltransferases encoding a NNMT_PNMT_TEMT domain in human, anole lizard, zebrafish, *Xenopus tropicalis* and *P. waltl*. Note the expansion of this family in *Xenopus* and *P. waltl*. Asterisks mark *P. waltl* genes that are specifically expressed in appendages and highlighted in Fig 2e. Phylogeny based on clustalo multiple alignments with 100 iterations.

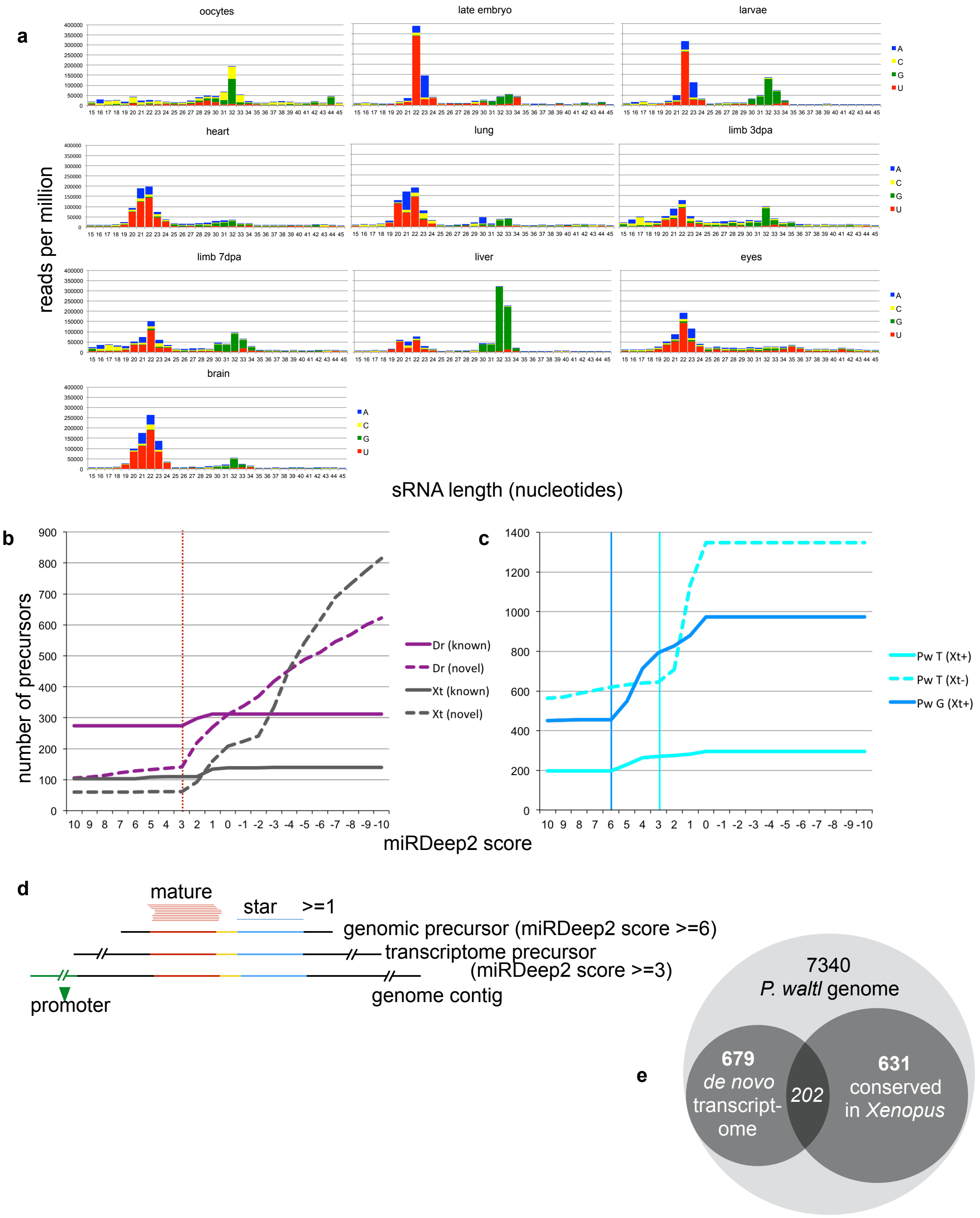

Supplementary Figure 3 | *P. waltl* miRNA sequencing and prediction. a, Histograms of small RNA length distribution with the initial nucleotide identified. Peaks at 22 nt and 32 nt are miRNAs and tRNA fragments, respectively. **b,** The increase of predicted novel and detected

known miRNAs using published *X. tropicalis* and zebrafish datasets. As miRDeep2 score cutoff decreases, the number of novel predictions increases at a rate higher than the rate of detecting known miRNAs. **c,** A cutoff score of 3 was adopted for *P. waltl* transcriptome predicted miRNA precursors based on miRDeep2 performance with *X. tropicalis* and zebrafish datasets. However, a more stringent score of 6 was chosen for genomic precursors due to the rapid increase in predicted precursors below that score. **d-e**, Small RNAs were purified from 8 different body parts and two limb regeneration time points. Libraries made with TruSeq small RNA were sequenced. miRDeep2 identified 23,425 genomic regions that exhibit a signature characteristic of miRNA precursors (i.e. transcribed RNA can form a hairpin and small RNA reads between 19 and 24 predominantly map to one of the hairpin arms). 7,340/23,425 predictions correspond to precursors with a score greater than 6, at least 1 read mapped to the star strand, passed the random fold analysis, and did not have any rfam alerts. 361/7,340 precursors had mature miRNA seeds conserved in *Xenopus*. miRDeep2 also identified 1,621 precursors embedded in *de novo* assembled transcripts. 679/1,621 predictions correspond to precursors with a score greater than 3, at least 1 read mapped to the star strand, passed the random fold analysis, and did not have any rfam alterts. 248/679 precursors had mature miRNA seeds conserved in *Xenopus*. These 248 precursors were present in 202 transcripts, which were included in the reference for gene expression quantification.

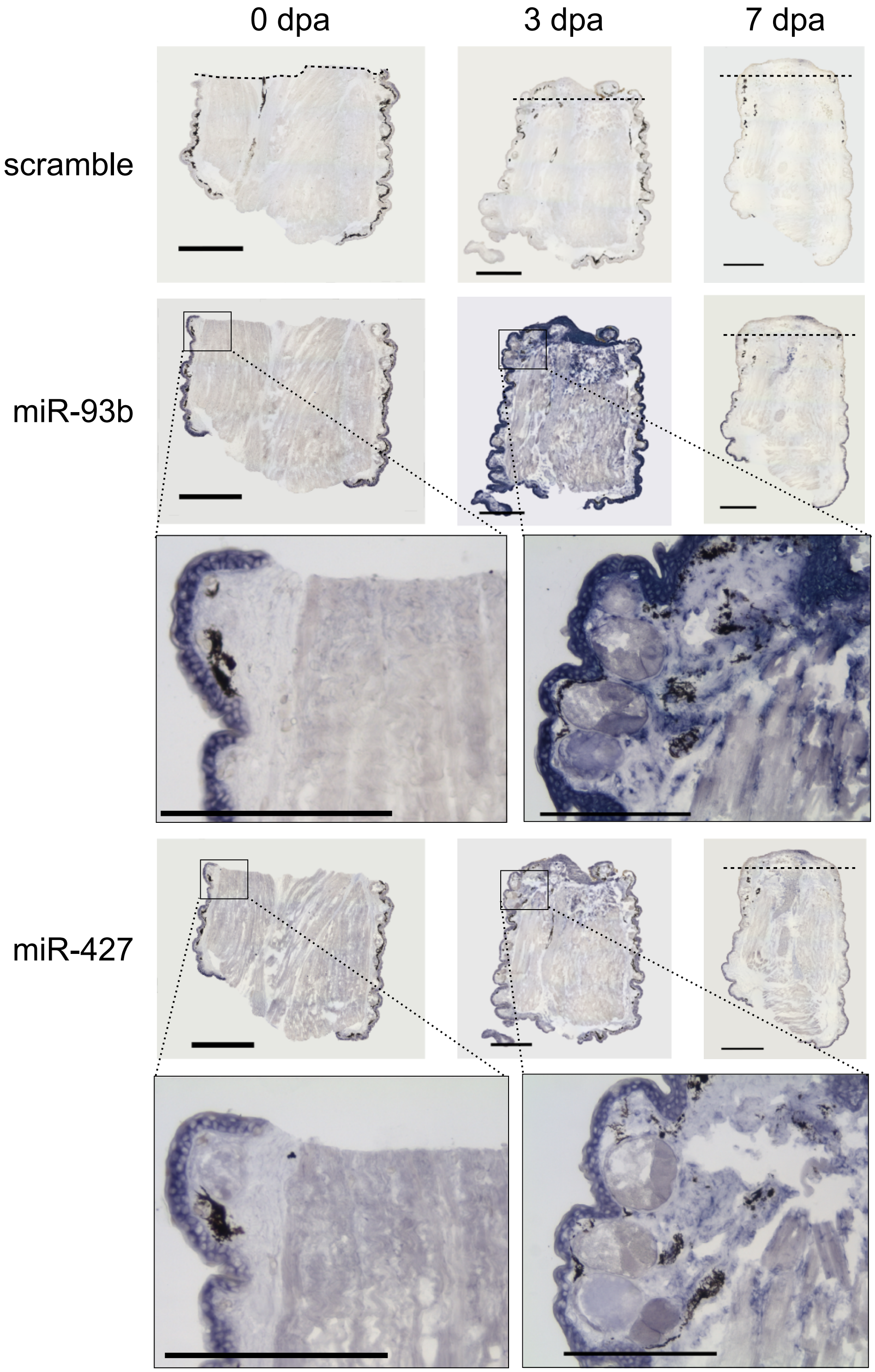

Supplementary Figure 4 | *In situ* hybridization visualizing upregulation of miR-93b and miR-427 in regenerating limbs. Digoxygenin-labeled Locked Nucleic Acid (LNA) probes

against the mature form of miR-93b and miR-427 in forelimb sections 0, 3, and 7 days post amputation (dpa). miR-93b and miR-427 signal increase at 3 dpa before falling at 7 dpa. All scale bars represent 0.5 mm for whole section and 0.25 mm for insets. Dashed lines mark amputation plane.

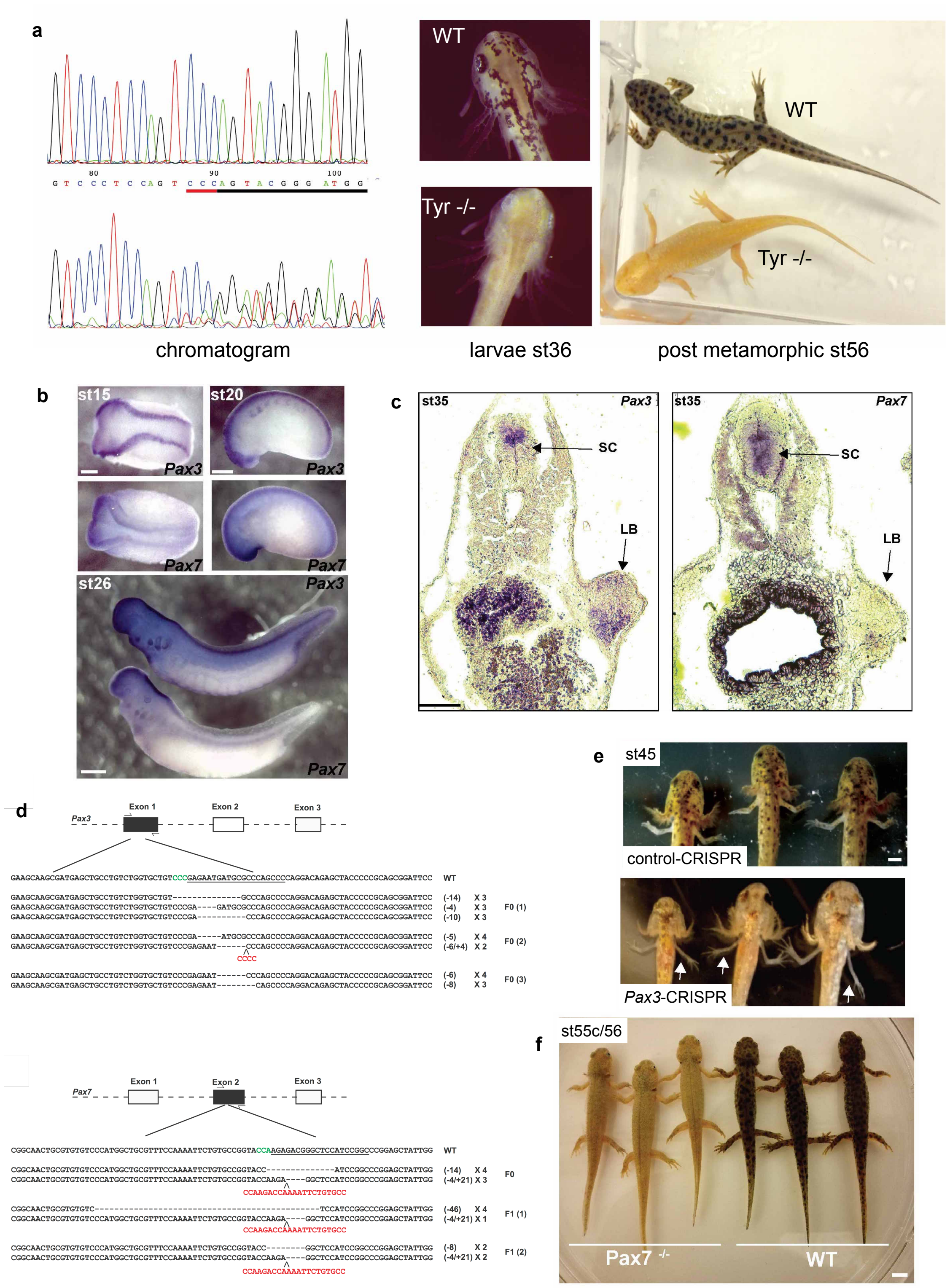

Supplementary Figure 5 | *P. waltl Pax3* and *Pax7* are retained and functional. a, Albino *P. waltl* were generated by knocking out the *Tyrosinase* gene (*Tyr*) with CRISPR/Cas9. (left)

Chromatogram showing mutation of *Tyrosinase* locus corresponding to guide sequence (black line, red line = PAM site). **b,** *Pax3* and *Pax7* whole-mount *in situ* hybridization of albino *P. waltl* embryos at stage 15, 20 and 26. Note the partially overlapping but distinguishable expression of *Pax3* and *Pax7* in neural tube and spinal cord at all stages analyzed. *Pax3*, but not *Pax7*, is also expressed in somites at stage 20. Scale bars = 500 µm. **c,** *Pax3* and *Pax7 in situ* hybridization of *P. waltl* larvae, transverse sections at the upper trunk level including the limb bud. *Pax3* is expressed in the dorsal spinal cord (SC) and the limb bud (LB). *Pax7* is expressed in the dorsal field of the spinal cord but not in the limb bud. Scale bar = 500 µm. **d,** (top) Genomic sequence of *Pax3*-CRISPR/Cas9 injected animals (F_0). (bottom) Genomic sequence of *Pax7*-CRISPR/Cas9 injected animals (F_0) and *Pax7*$^{-/-}$ knockout animals (F_1). The *Pax7*$^{-/-}$ knockout animals (F_1) were produced from mating of *Pax7*-CRISPR/Cas9 injected animals (F_0). **e,** Representative pictures of control-CRISPR/Cas9 and *Pax3*-CRISPR/Cas9 larvae. Arrows point to disabled limbs in *Pax3*-CRISPR/Cas9 animals. Scale bar = 1 mm. **f,** Representative pictures of *Pax7* $^{-/-}$ (F_1) and wild-type animals of the same developmental stage (post-metamorphic stage 55c/56). Note the pigmentation defect in *Pax7*$^{-/-}$ animals. Scale bar = 5 mm.

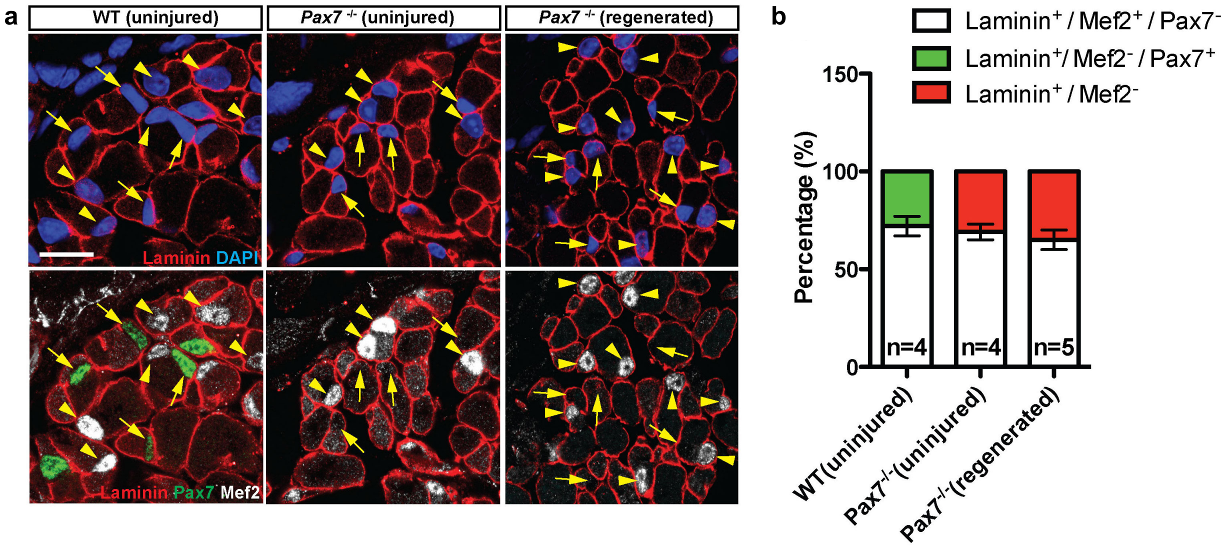

Supplementary Figure 6 | Post-metamorphic muscle regeneration is *Pax7* independent. a, Satellite cells are identified by $Pax7^{+}Mef2^{-}Laminin^{+}$ muscle nuclei. $Pax7^{+}$ satellite cell nuclei and $Mef2^{+}$ myonuclei are mutually exclusive in skeletal muscle. Both $Pax7^{+}$ satellite cell nuclei and $Mef2^{+}$ myofiber nuclei are under skeletal muscle basal lamina (Laminin). Neither $Pax7^{+}Mef2^{+}$ nor $Pax7^{-}Mef2^{-}$ cells are found under the basement membrane. Hence $Mef2^{-}$ $Laminin^{+}$ nuclei are $Pax7^{-}$ satellite cell nuclei in the skeletal muscle of *Pax7*$^{-/-}$ animals. The loss of *Pax7* does not alter the distribution and number of $Mef2^{-}Laminin^{+}$ nuclei (satellite cell nuclei) in the *Pax7*$^{-/-}$ animals. Arrows point to $Mef2^{-}Laminin^{+}$ muscle nuclei. Arrowheads point to $Mef2^{+}Laminin^{+}$ myonuclei. Scale bar represents 10 µm **b,** Quantification of $Pax7^{-}$ satellite cells ($Mef2^{-}Laminin^{+}$) in the skeletal muscle before and after limb regeneration in *Pax7*$^{-/-}$ animals. The number of $Pax7^{-}$ satellite cells remains comparable in uninjured and regenerated (45dpa) skeletal muscle of *Pax7*$^{-/-}$ animals. Error bars represent s.e.m.

Supplementary Table 1 |Statistics of the *P. waltl* genome

P. waltl Genome	
Size (1*n*)	19.38 Gb
Karyotype	2*n* = 24
Assembly	
Length (bp)	19,256,400,971
Number of fragments	65,627,701
% in range <10^2/10^2-10^3/10^3-10^4/10^4-10^5/>10^5 bp	12/36/43/5/4%
Size in fragments >1 kb/>10 kb	10Gb/1.7Gb
Shortest/longest fragment	71bp/7,104,246bp
N50	1.136 kb
TE landscape (based on repeats > 1kb)	
Gypsy	37.9%
Harbinger	27.6%
piggyBack/DIRS/L1/hAT/ERV/RTE/Helitron/Copia	10.6/6.6/5.6/4.8/3.5/1.6/1.4/0.4%
Annotation	
Genes and gene-like fragments	79,916
Protein-coding gene models	14,805
miRNAs	361
P. waltl Transcriptome	
Number of transcripts	6,440,242
gene level contigs encoding complete ORFs	98,628
transcripts encoding complete ORFs	193,464
Orthology groups	19,903
Groups with *P. waltl* gene models	10,666
Groups with *P. waltl* transcripts and no gene models	9,237
Vertebrate groups	17,198
Amphibian groups	1,130
Salamander groups	1,575

Supplementary Table 2 | Illumina genome sequencing output.

Lane	Million read pairs
1	303,4
2	304,3
3	306,0
4	306,3
5	307,0
6	265,4
7	267,8
8	267,5
9	268,4
10	269,1
Total	2865,2

Supplementary Table 3 | *P. waltl* samples used for RNAseq and reads obtained. Samples in bold were used for the transcriptome assembly.

Lane	ID	Description	BarcodeSeq	MSequenced
**1**	**P2024_2001**	**eyes**	**CGATGT**	**107,82174**
**1**	**P2024_2002**	**lung**	**TGACCA**	**79,346228**
1	P2024_2003	soft tissue	ACAGTG	69,475291
**1**	**P2024_2004**	**oocytes**	**CTTGTA**	**58,8802**
**2**	**P2024_2005**	**larvae (limb bud stage)**	**TTAGGC**	**81,421367**
**2**	**P2024_2006**	**brain**	**GAGTGG**	**69,319847**
**2**	**P2024_2007**	**liver**	**ACTGAT**	**95,264409**
**2**	**P2024_2008**	**heart**	**ATTCCT**	**75,044616**
**3**	**P2024_2009**	**distal tail**	**GCCAAT**	**34,020672**
**3**	**P2024_2010**	**hindlimbs**	**CAGATC**	**58,316309**
**3**	**P2024_2011**	**forelimbs**	**ATGTCA**	**62,568394**
3	P2024_2012	regenerating fore limb 3dpa A	GTCCGC	50,378898
**3**	**P2024_2013**	**late embryo (s22 – s25)**	**GTGAAA**	**44,665553**
**3**	**P2024_2014**	**regenerating forelimb 7dpa A**	**GATCAG**	**63,482349**
4	P2024_2015	forelimb stump 0dpa A	AGTCAA	14,851065
4	P2024_2016	forelimb stump 0dpa B	AGTTCC	7,970995
4	P2024_2017	forelimbs	ATGTCA	20,373158
4	P2024_2018	regenerating forelimb 3dpa B	CCGTCC	10,145021
**4**	**P2024_2019**	**regenerating forelimb 3dpa C**	**GTCCGC**	**26,084436**
4	P2024_2020	late embryo (s22 – s25)	GTGAAA	12,145227
4	P2024_2022	larvae (limb bud stage)	TTAGGC	29,254093
4	P2024_2024	regenerating forelimb 7dpa B	GATCAG	34,264054
4	P2024_2025	regenerating forelimb 7dpa C	TAGCTT	23,728724

Supplementary Table 4 | Statistics for Trinity *de novo* assembly of *P. waltl* transcriptome.

	ALL transcript contigs	LONGEST ISOFORM per 'GENE'
Total	6,440,242	5,555,520
Contig N10	4042	3228
Contig N20	2576	1939
Contig N30	1738	1272
Contig N40	1182	875
Contig N50	806	626
Median contig length	326	316
Average contig	580.68	516.33
Total assembled bases	3,739,725,420	2,868,503,231

Supplementary Table 5 | CEGMA and BUSCO completeness validations for *P. waltl* genome and transcriptome.

Sequence	Test	Complete	Fragmented	Missing	Number

		[Duplicated]	[Partial]		
P. waltl genome	CEGMA	59.68%[n/a]	n/a[93.55%]	n/a	458
	BUSCO-e	44% [1.3%]	12%	42%	429
	BUSCO-m	64% [1.7%]	7.3%	28%	843
	BUSCO-v	20% [0.5%]	16%	62%	3023
H. sapiens genome	CEGMA	97.58%[n/a]	n/a [100.00%]	n/a	458
	BUSCO-e	64% [7.4%]	7.6%	27%	429
	BUSCO-m	86% [5.3%]	2.7%	10%	843
	BUSCO-v	32% [0.8%]	26%	40%	3023
H. vulgare genome	CEGMA	87.10%[n/a]	n/a [98.79%]	n/a	458
	BUSCO-e	42% [7.9%]	13%	44%	429
	BUSCO-m	15% [3.7%]	7.2%	77%	843
	BUSCO-v	1.4%[0.3%]	1.0%	97%	3023
P. abies genome	CEGMA	54.03%[n/a]	n/a [84.08%]	n/a	458
	BUSCO-e	25% [5.8%]	17%	56%	429
	BUSCO-m	13% [3.6%]	6.6%	79%	843
	BUSCO-v	2.3% [0.4%]	0.8%	96%	3023
X. laevis genome	CEGMA	96.77%[n/a]	n/a [99.60%]	n/a	458
	BUSCO-e	72% [31%]	2.7%	24%	429
	BUSCO-m	91% [42%]	1.6%	6.8%	843
	BUSCO-v	32% [14%]	20%	46%	3023
X. tropicalis genome	CEGMA	93.15%[n/a]	n/a [96.77%]	n/a	458
	BUSCO-e	68% [1.3%]	5.3%	26%	429
	BUSCO-m	87% [2.2%]	3.3%	9.4%	843
	BUSCO-v	30% [0.3%]	21%	48%	3023
P. waltl transcriptome	CEGMA	93.95%[n/a]	n/a[100.00%]	n/a	458
	BUSCO-e	82%[68%]	11%[n/a]	6.5%	429
	BUSCO-m	88%[73%]	9.7%[n/a]	1.3%	843
	BUSCO-v	82%[64%]	9.2%[n/a]	8.1%	3023
N. viridescens transcriptome	CEGMA	99.19%[n/a]	n/a [100.00%]	n/a	458
	BUSCO-e	92%[88%]	2.0%	5.3%	429
	BUSCO-m	97%[92%]	1.7%	0.2%	843
	BUSCO-v	92%[78%]	2.6%	4.6%	3023

BUSCO-e (eukaryota set), -m (metazoa set) and -v (vertebrata set). Complete (and Duplicated), Fragmented (or Partial) and Missing refer to the state of the gene set used to estimate completeness. n/a refers to analyses not conducted by one algorithm (i.e. CEGMA or BUSCO) but not the other.

Supplementary Table 6 | Statistics of individual and merged *N. viridescens* transcriptomes

	Looso *et al.*[2]	Abdullayev *et al.*[3]	regenerating limb	merged
length (bp)	73,541,735	199,694,626	300,090,706	380,044,646
# of sequences	120,922	132,861	463,798	431,864
N25 stats (bp)	2,151	4,727	2,094	3,156
N50 stats (bp)	975	2,315	692	1,297
N75 stats (bp)	486	986	388	564
GC %	44.12	45.38	43.57	44.26

Supplementary Table 7 | *P. waltl* samples used for small RNAseq and reads obtained.

NGI ID	User ID	Million reads	Barcode	>=Q30(%)
P3761_2001	eyes	58.8	AGTCAA	98.63
P3761_2002	heart	42.69	AGTTCC	98.69
P3761_2003	lung	10.87	ATGTCA	98.68
P3761_2004	oocytes	7.85	CCGTCC	98.53
P3761_2005	forelimb 3dpa	44.1	GTAGAG	98.67
P3761_2006	late embryo	11.68	GTCCGC	98.71
P3761_2007	larvae	36.22	GTGAAA	98.7
P3761_2008	forelimb 7dpa	25.96	GTGGCC	98.63
P3761_2009	brain	20.58	GTTTCG	98.7
P3761_2010	liver	29.26	CGTACG	98.6

Supplementary Table 8 | Published small RNA data used for miRDeep2 prediction of *X. tropicalis* and zebrafish miRNAs.

Organism			Description	Ref.
zebrafish	GSM1376617	SRR1265734	embryo	[4]
zebrafish	GSM1376620	SRR1265737	male brain	[4]
zebrafish	GSM1376623	SRR1265740	female brain	[4]
zebrafish	GSM1376626	SRR1265743	male gut	[4]
zebrafish	GSM1376629	SRR1265746	female gut	[4]
zebrafish	GSM1376632	SRR1265749	male liver	[4]
zebrafish	GSM1376635	SRR1265752	female liver	[4]
zebrafish	GSM1376638	SRR1265755	ovary	[4]
zebrafish	GSM1376641	SRR1265758	testis	[4]
zebrafish	GSM1376644	SRR1265761	eye	[4]
zebrafish	GSM1376647	SRR1265764	heart	[4]
X. tropicalis	GSM945997	SRR505561	stage 8	[5]
X. tropicalis	GSM945998	SRR505562	stage 10	[5]
X. tropicalis	GSM945999	SRR505563	stage 18	[5]
X. tropicalis	GSM946000	SRR505564	stage 10 Animal Pole	[5]
X. tropicalis	GSM946001	SRR505565	stage 10 Vegetal Pole	[5]
X. tropicalis	GSM744254	SRR285187	gastrula ventral explant	[6]
X. tropicalis	GSM744253	SRR285186	gastrula dorsal explant	[6]
X. tropicalis	GSM372598	SRR020456	adult liver	[7]
X. tropicalis	GSM372601	SRR020457	adult skin	[7]
X. tropicalis	GSM372602	SRR020458	oocytes stage I, II	[7]
X. tropicalis	GSM372603	SRR020459	oocytes stage III, IV	[7]
X. tropicalis	GSM372604	SRR020460	oocytes stage V, VI	[7]

Supplementary Table 9 | mir-93b, mir-427 primary transcripts, five Harbinger (*Myb* and *Harbi* containing) orthology groups and four Gypsy (reverse transcriptase containing) orthology groups are upregulated during limb regeneration (3dpa).

Description	id	log2FoldChange	lfcSE	stat	pvalue	padj
Myb	BBL002186	-2.393	0.345	-6.931	4.177e-12	1.121e-10
Myb	BBL014413	-1.535	0.339	-4.528	5.949e-06	5.191e-05
Myb	BBL000953	-2.356	0.336	-6.994	2.658e-12	7.447e-11
Harbi	BBL000620	-1.589	0.329	-4.820	1.431e-06	1.468e-05
Harbi	BBL000792	-1.635	0.327	-4.985	6.185e-07	6.848e-06
Gypsy	BBL001700	-1.138	0.319	-3.563	0.00036	0.00200
Gypsy	BBL019848	-1.714	0.372	-4.600	4.208e-06	3.825e-05
Gypsy	BBL025685	-1.395	0.366	-3.809	0.00013	0.00086
Gypsy	BBL042907	-1.858	0.358	-5.186	2.145e-07	2.603e-06
miRNA	pri-mir-427	-1.599	0.317	-5.040	4.631e-07	1.825e-05
miRNA	pri-mir-93b	-1.301	0.318	-3.851	0.00011	0.00227
Mtransase	BBL019842	1.508	0.086	13.57	5.661e-42	8.182e-40
Mtransase	BBL021312	1.603	0.252	6.361	1.995e-10	2.138e-09
Mtransase	BBL013342	1.171	0.239	6.300	2.974e-10	3.108e-09
Mtransase	BBL033310	2.145	0.341	6.288	3.208e-10	3.335e-09

Four NNMT_PNMT_TEMT salamander methyltransferases are appendage specific and downregulated during limb regeneration (7dpa). Analysis via DESeq2 comparing uninjured forelimb versus forelimb 3dpa or 7dpa where negative log2FC corresponds to an increase in 3dpa and positive log2FC corresponds to decrease in 7dpa. The Orthology group names starting with BBL are in the same order as depicted in Fig. 2e and rows 2, 7, 8 and 11 in Fig. 2f. lfcSE = log2FoldChange standard error, stat = Wald statistic, padj = adjusted p-value.

Supplementary Table 10 | Low *Naif1* and *Harbi1* expression (TPM) compared to other Harbinger transposable elements that are regulated during limb regeneration.

	Naif1 BBL016369	*Harbi1* BBL012737	BBL002186	BBL014413	BBL000953	BBL000620	BBL000792
oocyte	7.619	7.754	6.444	12.254	5.426	77.645	18.930
lateemb	3.214	2.611	184.247	205.262	125.034	430.465	178.329

lateemb	1.568	2.008	177.320	200.152	111.034	401.490	186.781
larvae	1.396	4.167	476.737	558.758	323.586	1069.616	436.915
larvae	2.113	4.277	456.108	636.971	325.460	1065.785	455.186
eyes	1.425	2.994	3226.582	2659.944	1622.688	3648.455	1709.528
lung	2.413	4.916	525.786	452.166	197.185	668.886	269.715
softtissue	3.663	6.333	222.762	248.128	98.478	330.954	128.148
brain	0.967	2.385	3479.394	4587.396	2917.581	7545.221	3324.371
liver	1.311	2.581	992.031	1237.460	773.502	2129.631	945.358
heart	0.992	2.663	726.261	962.414	539.474	1789.645	829.500
forelimb	1.878	3.607	3789.821	2547.467	1267.651	3174.313	1441.038
forelimb	1.855	3.435	1303.179	987.485	448.550	1330.205	572.105
forelimb	1.451	2.731	2121.309	1466.238	773.598	1639.855	759.085
forelimb	2.108	2.836	2025.332	1359.625	711.225	1620.073	692.214
r3dpa	2.223	3.006	10687.697	3754.112	3711.679	5075.031	2424.639
r3dpa	2.609	3.865	19392.956	7358.940	6472.103	9129.109	4145.149
r3dpa	2.476	3.476	10991.027	3949.333	3790.200	5103.470	2435.030
r7dpa	1.903	3.128	1949.089	1000.551	1110.321	1615.158	607.399
r7dpa	1.636	3.111	2019.978	1063.226	1115.669	1682.468	615.844
r7dpa	1.720	2.526	937.531	950.766	737.388	1502.317	570.061
hindlimb	2.399	2.226	7135.884	2964.593	2013.275	3081.215	1419.051
tail	1.915	1.923	2337.120	1464.637	657.949	1207.228	550.120

Supplementary Table 11 | Primers and gRNA sequences for CRISPR/Cas9

Primer name	Sequence
Tyrosinase gRNA	GAACTTCACCATCCCGTACT
Pax3 gRNA	GGGCTGGGCGCATCATTCTC
Pax7 gRNA	GCCGGATGGAGCCCGTCTCT
Tyrosinase genotyping forward	CGACTTCGCACACGAGG
Tyrosinase genotyping reverse	CTGCCAGGAGGAGAAGAATG
Pax3 genotyping forward	TGTGCTGGGACAGGGACTT
Pax3 genotying reverse	ATTCTGTAGGCGCAGTAAGTAT
Pax7 genotyping forward	TCCGCCACAAGATCGTGGAGATG
Pax7 genotyping reverse	TTGCTGCCCCCAATAGCT
Pax3 probe forward	CCAGGGTCGAGTGAACCAACT
Pax3 probe reverse	TTCCTCCCGTGTGTAGATAT
Pax7 probe forward	CACAGCTTCTCCAGTTACTC
Pax7 probe reverse	TTGCCTTGGAGTGCGTCAGTA

Supplementary Data 1 (separate file)

Orthology groups. Sheet1: Groups are prefixed with BBL and the number of members from each species is indicated (hsa = *H. sapiens*, mmu = *M. musculus*, gga = *G. gallus*, aca = *A. carolinensis*, xla = *X. laevis*, xtr = *X. tropicalis,* dre = *D. rerio*, ame = *A. mexicanum*, nvi = *N. viridescens*, pwaT = *P. waltl* transcript and pwaG = *P. waltl* gene model). Groups are annotated based on the most frequent gene symbol appearing in the group. Groups with non-salamander members and no annotation are designated unknown-conserved. Groups with only salamander members and no detected Pfam domains are designated unknown-salamander. Note that columns nvi, ame and pwaT are based on transcriptome predicted proteins. All other columns including pwaG are based on gene-model predicted proteins. Sheet 2: The 19,903 groups including a *P. waltl* orthologs and their classification.

Supplementary Data 2 (separate file)

Conversion table for TransDecoder and Maker to OrthoMCL compatible id headers (hsa = *H. sapiens*, mmu = *M. musculus*, gga = *G. gallus*, aca = *A. carolinensis*, xla = *X. laevis*, xtr = *X. tropicalis,* dre = *D. rerio*, ame = *A. mexicanum*, nvi = *N. viridescens*)

Supplementary Data 3 (separate file)

Conversion table for TransDecoder and Maker to OrthoMCL compatible id headers (pwa = *P. waltl*)

Supplementary Data 4 (separate file)

P. waltl miRNAs. miRDeep2 calibration runs with *Xenopus* and zebrafish datasets, genome predictions, transcriptome predictions, mature miRNA quantification and the top 10 miRNAs upregulated in the regenerating tissue (limbs 3dpa and 7dpa) compared to the uninjured adult tissue (brain, eyes, heart, liver, lung).

Supplementary Movie 1 (separate file)

P. waltl Pax3 CRISPR/Cas9 mosaic knockout larva with muscle-less forelimb (right).

Supplementary Methods:

Illumina data processing

Illumina Paired End (IPE) reads, from genomic DNA and RNA libraries, were quality filtered using the nesoni clip suit (https://github.com/Victorian-Bioinformatics-Consortium/nesoni.git). Sequencing adaptors, read bases with a per-base Phred quality value below 20 and reads shorter than 64 nucleotides were excluded.

```
nesoni clip --quality 20 --length 64 --homopolymers yes --out-separate
yes --gzip yes read_file.g.ipe.Q20L64H pairs: read_file_1.fq.gz
read_file_2.fq.gz
```

In the case that one of the two paired ends passed the quality filtered but not the counterpart, the read was kept and designated as an Illumina single end (ISE). Filtered IPE reads were merged with FLASH [8] and these merged pairs were treated as single end reads (ISE).

```
flash -z --compress-prog=pigz -t 16 -p 33 -r 120 -l 180 -s 80 -o
read_file.g.ipe.Q20L64H_FLASH read_file_1.fq.gz read_file_2.fq.gz
```

Genome assembly unitig construction

Genomic IPE reads were first assembled into unitigs using a modified implementation, in the Fermi assembler [9], of the previously described String Graph algorithm [10]. First, genomic IPE were error corrected and subsequently used to build a string graph, requiring a minimum overlap of 45 nucleotides between the IPE reads. After construction and data reduction, unitigs were extracted from this graph. These unitigs - referred here as Fermi-unitigs - were used for miRNA gene prediction and represent the first version of the P. waltl genome assembly, Pwaltl_v1.0.

```
perl run-fermi.pl -t 32 -e fermi -k 45 -p Pwaltl_v1.0 -P
read_file_1.fq.gz read_file_2.fq.gz > Pwaltl_v1.0.mak

make -f  Pwaltl_v1.0.mak -j 32
```

Genome assembly contig construction

Contigs were assembled using the IPE and ISE data with Abyss v1.9.0 (k = 71) [11]. Non-merged IPE were supplied as input along the merged IPE and ISE.

```
abyss-pe np=32 n=5 k=71 name=Pwaltl_v4.1 lib='pe1 pe2 pe3 pe4 pe5 pe6
pe7 pe8 pe9 pe10' se='se1 se2 se3 se4 se5 se6 se7 se8 se9 se10'
pe1='read_file_1.fq.gz read_file_2.fq.gz' pe2='read_file_1.fq.gz
read_file_2.fq.gz' pe3='read_file_1.fq.gz read_file_2.fq.gz'
pe4='read_file_1.fq.gz read_file_2.fq.gz' pe5='read_file_1.fq.gz
read_file_2.fq.gz' pe6='read_file_1.fq.gz read_file_2.fq.gz'
pe7='read_file_1.fq.gz read_file_2.fq.gz' pe8='read_file_1.fq.gz
read_file_2.fq.gz' pe9='read_file_1.fq.gz read_file_2.fq.gz'
pe10='read_file_1.fq.gz read_file_2.fq.gz'
se1='read_file.g.ipe.Q20L64H_FLASH_001.fq.gz'
se2='read_file.g.ipe.Q20L64H_FLASH_002.fq.gz'
se3='read_file.g.ipe.Q20L64H_FLASH_003.fq.gz'
se4='read_file.g.ipe.Q20L64H_FLASH_004.fq.gz'
se5='read_file.g.ipe.Q20L64H_FLASH_005.fq.gz'
se6='read_file.g.ipe.Q20L64H_FLASH_006.fq.gz'
se7='read_file.g.ipe.Q20L64H_FLASH_007.fq.gz'
se8='read_file.g.ipe.Q20L64H_FLASH_008.fq.gz'
se9='read_file.g.ipe.Q20L64H_FLASH_009.fq.gz'
se10='read_file.g.ipe.Q20L64H_FLASH_010.fq.gz'
```

The assembly was executed in a machine running Scientific Linux with 32 cores and 2 TiB of RAM at the Stockholm node of the Science for Life Laboratory, for a total duration of 196 hours and a peak RAM memory usage of 1.6 TiB. This strategy generated assembly Pwaltl_v4.1. These contigs were afterwards used as input for scaffolding with RNAseq transcripts.

Genome assembly transcript contig construction

RNAseq data were assembled into transcripts using all the transcriptomic tissue libraries with Trans-Abyss v1.5.4 [12]. A K-mer size of 69 was used to improve the reconstruction of common and repetitive transcripts over rare transcripts; thus discrete isoforms and short RNAs may not be represented in this transcriptome assembly.

```
transabys --threads 16 --kmer 69 --cov 5 --length 300 –pe
read_file.rna.ipe.Q20L64H_XXX_R1.fastq
read_file.rna.ipe.Q20L64H_XXX_R2.fastq
```

where XXX represents the RNAseq library barcode.

Genome assembly contig scaffolding

The Pwaltl_v4.1 assembly was scaffolded using assembled transcripts with the aim to improve the reconstruction of gene content. These RNAseq ordered contigs are referred here as scaffolds. The derived RNAseq scaffolded assembly was designated as Pwaltl_v4.2.

```
abyss-pe np=32 n=5 k=71 name=Pwaltl_v4.2 lib='pe1 pe2 pe3 pe4 pe5 pe6
pe7 pe8 pe9 pe10' se='se1 se2 se3 se4 se5 se6 se7 se8 se9 se10'
pe1='read_file_1.fq.gz read_file_2.fq.gz' pe2='read_file_1.fq.gz
read_file_2.fq.gz' pe3='read_file_1.fq.gz read_file_2.fq.gz'
pe4='read_file_1.fq.gz read_file_2.fq.gz' pe5='read_file_1.fq.gz
read_file_2.fq.gz' pe6='read_file_1.fq.gz read_file_2.fq.gz'
pe7='read_file_1.fq.gz read_file_2.fq.gz' pe8='read_file_1.fq.gz
read_file_2.fq.gz' pe9='read_file_1.fq.gz read_file_2.fq.gz'
pe10='read_file_1.fq.gz read_file_2.fq.gz'
se1='read_file.g.ipe.Q20L64H_FLASH_001.fq.gz'
se2='read_file.g.ipe.Q20L64H_FLASH_002.fq.gz'
se3='read_file.g.ipe.Q20L64H_FLASH_003.fq.gz'
se4='read_file.g.ipe.Q20L64H_FLASH_004.fq.gz'
se5='read_file.g.ipe.Q20L64H_FLASH_005.fq.gz'
se6='read_file.g.ipe.Q20L64H_FLASH_006.fq.gz'
se7='read_file.g.ipe.Q20L64H_FLASH_007.fq.gz'
se8='read_file.g.ipe.Q20L64H_FLASH_008.fq.gz'
se9='read_file.g.ipe.Q20L64H_FLASH_009.fq.gz'
se10='read_file.g.ipe.Q20L64H_FLASH_010.fq.gz'
long1='Pwaltl_mRNA.v.3.0.fasta'
```

The assembly of the axolotl *Ambystoma mexicanum* (150 MYA from newt, N50 = 3Mb) was used to arrange newt contigs along longer axolotl scaffolds. Pwaltl_v4.2 was first masked using RepeatMasker (`RepeatMasker -species amphibian`) and then aligned to the Ambystoma

genome using BLASTN (`-evalue 0.0001`). The alignments were used as input for Chromosomer[13]. The resulting assembly was denoted Pw_v5.0 and consisted of 581,351 mapped fragments forming 26,053 higher-level scaffolds of total length 959,236,654 bp.

This assembly was then concatenated with the remaining fragments of Pw_v4.2, which were not arranged along an *Ambystoma* scaffold, resulting in assembly Pw_v5.1. Finally, sequence ids were formatted as follows: Suffix – PWALG for *P. waltl* genome. First digit representing sequence length range (based on the number digits in length, i.e. 4 for 1,000 – 9,999 and 5 for 10000 – 99999, etc.). Second digit representing source of contig/scaffold (0 if created by scaffolding against Ambystoma genome, i.e. Pw_v5.0. 1-9 if from Pw_v4.2).

The remaining digits are copied from original id. If the sequence is based on Ambystoma scaffolding (Pw_v5.0), then this number is the same as the *Ambystoma* scaffold id and begins with 0. Otherwise, the remaining digits are the ones assigned by Abyss in Pw_v4.2 and start with 1 through 9.

For example, *P. waltl* sequence `PWALG60030000001` is within 100,000-999,999 bp range and originates from *Ambystoma* scaffolding step where sequence `AMEX_0030000001` served as a scaffold.

***P. waltl de novo* transcriptome assembly**

856,236,120 paired-end reads were assembled to a single reference transcriptome assembly (Supplementary Table 3) using Trinity version 2.1.0 with the paired-end reverse-forward mode. Reads matching adapter sequences were removed using the read trimming tool Trimmomatic and any reads shorter than 36 base pairs were excluded from further assembly. The coverage depth used to normalize the reads was 30x. Trinity assembly was divided in 3 steps as follows:

First, adapter-filtering and digital normalisation (flag "`--no_run_inchworm`"):

```
Trinity --seqType fq --SS_lib_type RF --max_memory 450G --CPU 16 --
min_kmer_cov 2 --inchworm_cpu 12 --bflyGCThreads 10 --bflyCPU 16 --
trimmomatic --quality_trimming_params "ILLUMINACLIP:TruSeq3-
SE_all_indexes.fa:2:30:10 MINLEN:36" --normalize_reads --
normalize_max_read_cov 30 --normalize_by_read_set --no_run_inchworm --
verbose --left $LEFT --right $RIGHT --output $OUT_TMP
```

Second, the inchworm step using the previous command after replacing `--no_run_inchworm` with `--no_run_chrysalis`.

Third, the chrysalis/butterfly step using the previous command without `--no_run_chrysalis`.

Failed commands were re-run individually with larger memory allocation.
The final transcriptome Pw_v3.1 consisted of 5,555,520 Trinity 'genes' and 6,440,242 'transcripts' of which 217,169 transcripts encoded a complete ORF predicted by TransDecoder[14]. 100,848 complete ORFs matched one of 14,771 proteins in Swiss-Prot by BLASTP (E value < 1e-5).

***P. waltl* transcriptome completeness validation**
BUSCO and CEGMA completeness validation were done as described above with the exception that the BUSCO command was modified for a transcriptome input (`-m trans`).

***N. viridescens* transcriptome merging and annotation**
Two published *N. viridescens* transcriptomes [2,3] and the regenerating limb transcriptome were merged using Corset (v1.4)[15].
First, Illumina RNAseq reads from all three studies were mapped to the three transcriptomes (looso, abdul, limb) using the following command template:

```
STAR --genomeDir ../transcriptomedir/  --runThreadN 16 --readFilesIn
../transcriptomedir/seqfile_1.fastq
../transcriptomedir/seqfile_2.fastq --outFileNamePrefix
seqfile_transcriptome_ --limitGenomeGenerateRAM 100000000000  --
runDirPerm All_RWX  --outFilterMultimapNmax 1000 --
outFilterMismatchNoverLmax 0.05 --alignIntronMax 1 --alignIntronMin 2
--scoreDelOpen -10000 --scoreInsOpen -10000 --alignEndsType EndToEnd
```

Next, SAM files were converted to BAM and the BAM files were input into Corset for clustering as follows:

```
corset
2026_looso_Aligned.bam,2026_abdul_Aligned.bam,2026_limb_Aligned.bam
```

```
2027_looso_Aligned.bam,2027_abdul_Aligned.bam,2027_limb_Aligned.bam
2028_looso_Aligned.bam,2028_abdul_Aligned.bam,2028_limb_Aligned.bam
2029_looso_Aligned.bam,2029_abdul_Aligned.bam,2029_limb_Aligned.bam
4d_looso_Aligned.bam,4d_abdul_Aligned.bam,4d_limb_Aligned.bam
4v_looso_Aligned.bam,4v_abdul_Aligned.bam,4v_limb_Aligned.bam
8v_looso_Aligned.bam,8v_abdul_Aligned.bam,8v_limb_Aligned.bam
8d_looso_Aligned.bam,8d_abdul_Aligned.bam,8d_limb_Aligned.bam
s1_looso_Aligned.bam,s1_abdul_Aligned.bam,s1_limb_Aligned.bam
s2_looso_Aligned.bam,s2_abdul_Aligned.bam,s2_limb_Aligned.bam
s3_looso_Aligned.bam,s3_abdul_Aligned.bam,s3_limb_Aligned.bam
s4_looso_Aligned.bam,s4_abdul_Aligned.bam,s4_limb_Aligned.bam
s5_looso_Aligned.bam,s5_abdul_Aligned.bam,s5_limb_Aligned.bam -g
limb3dpa,limb3dpa,limb7dpa,arm2elbow,diris4dlm,viris4dlm,viris8dlm,dir
is8dlm,brain,heartlung,liver,soft,torso -m 50
```

The script fetchClusterSeqs.py was used to extract contigs from the three transcriptomes and compile Nv_v3.1.fasta.

```
fetchClusterSeqs.py -i combined_transcriptomes.fasta -t
clustersofinterest_nl2.csv -o Nv_v3.1.fasta -c clusters.txt
```

Transcripts with 'comp', 'transcript' or 'TR' in their id originate from [2,3] or the regenerating limb transcriptome, respectively.

TransDecoder (v2.0.1)[14] was run against the merged transcriptome to predict open reading frames and their translated proteins.

Genome *ab initio* gene prediction with Augustus

Complete gene models were predicted *ab initio* with Augustus using the generic model of gene structure. Gene models that contained stop codons were discarded as were gene models that produced a peptide sequence shorter than 50 amino acids.

Genome annotation generation of evidence for gene calling

Hints were generated by mapping complete protein and nucleotide sequences from multiple databases. For protein models, the entire set of validated proteins from UniProtKB/Swiss-Prot, UniProtKB/TrEMBL and uniref90; and for nucleotide models, the complete set of nucleotide sequences from the NCBI RefSeq database were retrieved. Nucleotide and protein sequences were mapped back to the genome assembly using BLAT.

```
blat -noHead -minIdentity=95 Pwaltl_4.2.masked_second.fasta
refseq_r75.nt.fasta Pwaltl_v4.2sm_vs_refseq-r75.psl
```

```
blat -noHead -minIdentity=95 -t=dnax Pwaltl_4.2.masked_second.fasta -
q=prot uniprot_sprot.aa.fasta Pwaltl_v4.2sm_vs_sprot.psl
```

The output PSL files were processed with the Augustus utilities to keep those alignments that had a minimal percentage of coverage of the query read above 85% and a minimum sequence identity of 95%.

```
cat Pwaltl_v4.2sm_vs_refseq-r75.psl | filterPSL.pl --best --
minCover=85 > Pwaltl_v4.2sm_vs_refseq-r75.f.psl
```

```
cat Pwaltl_v4.2sm_vs_sprot.psl | filterPSL.pl --best --minCover=85 >
Pwaltl_v4.2sm_vs_sprot.f.psl
```

RNAseq reads from the different tissue transcriptomic libraries of *P. waltl* were mapped against the genome assembly using the STARlong mapper in a machine with 32 cores and 2 TiB of RAM. The BAM files were processed with the bam2hints Augustus utility to obtain exon and intron hints from the RNAseq alignments while removing redundant alignments. Exons or intron features with more than 15 RNAseq hints were discarded for speed.

```
bam2hints --nomult --remove_redundant --maxcoverage=15 --intronsonly -
-in=larvae.RNAseq.sorted.bam --out=larvae.RNAseq.introns.gff
```

```
bam2hints --nomult --remove_redundant --maxcoverage=15 --
in=larvae.RNAseq.sorted.bam --out=larvae.RNAseq.exons.gff
```

The assembled transcripts used for the scaffolding step were filtered for transcripts shorter than 150 nucleotides in length and clustered using CD-HIT-EST using a minimum sequence identity of 90%.

```
cd-hit-est -i Pwaltl_mRNA.v.3.0.fasta -l 100 -c 0.9 -n 10 -M 64000 -T
16 -o Pwaltl_mRNA.v.3.0.clustered.fasta
```

These sequence clusters were later mapped to the genome using Exonerate.

```
exonerate --showquerygff yes --model est2genome
Pwaltl_mRNA.v.3.0.clustered.fasta Pwaltl_4.2.masked_second.fasta
```

Genome annotation hinted gene prediction with Augustus

Hints from external databases and the *ab initio* gene predictions were used as evidence for Augustus to call more accurate gene models. As before, the generic gene model was used to identify complete coding sequences.

```
augustus --species=generic --genemodel=complete --noInFrameStop=true -
-gff3=on --hintsfile=Pw_combined_hints.gff --
extrinsicCfgFile=extrinsic.MEP.cfg Pwaltl_4.2.masked_second.fasta >
Pwaltl_4.2.masked_second_Hinted.gff
```

Genes that contained stop codons within their coding sequences or produced a peptide shorter than 50 amino acids were discarded.

Orthology groups using OrthoMCL

The set of predicted *P. waltl* gene models and their translated proteins, in addition to the *de novo* assembled transcriptomes and predicted proteomes of salamanders *P. waltl*, *N. viridescens* (Methods and Materials) and *A. mexicanum* [14] were procured. To identify orthologs with other vertebrates, we procured the proteomes of *H. sapiens*, *M. musculus*, *G. gallus*, *A. carolinensis*, *X. tropicalis* and *D. rerio* from Ensembl.org in addition to that of *X. laevis* (XenBase.org).

Proteome files used: Homo_sapiens.GRCh38.pep.all.fa, Mus_musculus.GRCm38.pep.all.fa, Gallus_gallus.Gallus_gallus-5.0.pep.all.fa, Anolis_carolinensis.AnoCar2.0.pep.all.fa, Xenopus_tropicalis.JGI_4.2.pep.all.fa, Xenla_6.0_longest.pep.fa, Danio_rerio.GRCz10.pep.all.fa, Axolotl.Trinity.CellReports2017.transdecoder.pep, Nv_v3.1. transdecoder.pep, Pw_v3.1. transdecoder.pep and pw_v4.2_1kb.all.maker.proteins.fasta. Header ids were converted from TransDecoder and ENSEMBL conventions to `gsp|12345678`, where 'g' is the first letter of the genus and 'sp' the first two letters of the species, followed by the pipe symbol and eight digits. Conversion files between old TransDecoder or Maker and the new OrthoMCL compatible ids are provided as Supplementary Table 3. ENSEMBL ids were converted by taking the 8 digits to the left of the decimal (i.e. ENSP00000488424.1 to hsa|00488424 and ENSDARP00000002544.8 to dre|00002544); a conversion that did not lead to any duplicate ids and that allows easy conversion back to the original ENSEMBL ids. *P. waltl* proteins predicted from gene models start with `pwa|1`, while proteins predicted from de novo assembled transcripts start with `pwa|0`. The 11 proteomes were combined and used as input for OrthoMCL.

The output of OrthoMCL is the file groups.txt in which orthologus proteins are preceeded by a unique identifier. For example, the orthogroup for tumor suppressor retinoblastoma (RB1) is:

```
BBL007139: aca|00003415 ame|00029380 ame|00029381 ame|00029383
ame|00029385 dre|00021737 gga|00035704 gga|00063247 hsa|00267163
mmu|00022701 nvi|00091403 nvi|00091404 nvi|00091406 nvi|00091407
nvi|00091408 nvi|00091410 pwa|00132586 pwa|10035310 xla|00021595
xtr|00058442 xtr|00062636 pwa|00287550 pwa|00287551 xla|00021617
```

We classified RB1 as a "core vertebrate" orthology group since it consists of members from all 7 non-salamander groups (hsa = human, mmu = mouse, gga = chicken, aca = lizard, xla = *X. laevis*, xtr = *X. tropicalis*, dre = zebrafish). In addition the group includes 4 *A. mexicanum* transcripts, 6 *N. viridescens* transcripts, 3 *P. waltl* transcripts and 1 *P. waltl* gene model (`pwa|10035310`).

A group was designated 'vertebrate' if it included a non-amphibian component and 'amphibian' if it included a *Xenopus* component but no non-amphibian orthologs. Groups composed of only

salamander orthologs were designated 'salamander' if they included both a *P. waltl* gene model and de novo assembled transcript and had at least 1 member with a detected Pfam domain (hmmerscan evalue < 1e-04).

In total, OrthoMCL produced 118,205 groups, of which 8,259 were classified core vertebrate, 32,210 as vertebrate, 2,770 as amphibian and 1,575 as salamander. The groups that included a P. waltl member were as follows 8,108/8,259 core vertebrate, 17,198/32,210 vertebrate, 1,130/2,770 amphibian, and 1,575/1,575 salamander.

For gene expression analyses, open reading frames of the *P. waltl* transcripts and gene models were extracted from 17,198 vertebrate, 1,130 amphibian and 1,575 salamander orthogroups (19,903 groups total). In the RB1 example above, this means that ORFs of transcripts `pwa|00132586`, `pwa|00287550`, `pwa|00287551` and gene model `pwa|10035310` were included in the reference and represent RB1. The reference therefore consisted of 123,518 sequences (14,805 gene models and 108,713 transcripts) representing 19,903 orthogroups, in addition to 202 miRNA primary transcripts (Materials and Methods).

Expansion or contraction of orthologs was determined by analyzing orthology groups with at least three non-salamander members. Non-salamander species contributing to an orthology group define the range of orthologs within a given group (max and min).
Contraction (i.e. loss) was calculated by subtracting the orthology group's minimum value from the number of *P. waltl* gene models (pwaG) (after adding 2 to the number of *P. waltl* genes in order to avoid null values and account for incompleteness of gene set) and dividing the difference by the minimum value (after adding 1 to avoid null values).
Expansion was calculated by subtracting the number of *P. waltl* gene models from the orthology group's maximum value and dividing the difference by the maximum value (after adding 1 to avoid null values).

$$\text{Contraction} = (\text{pwaG}+2) - \text{min}/(\text{min}+1)$$

$$\text{Expansion} = \text{pwaG} - \text{max} / (\text{max}+1)$$

For example, the retinoblastoma protein RB1 belongs to orthogroup BBL007139, which in turn has a single member from human, mouse, lizard and zebrafish proteomes and two members from chicken, *X. laevis* and *X. tropicalis* proteomes. Therefore, the minimum number of group

members is 1 and the maximum is 2. In this case, the contraction and expansion values would equal:

$$\text{Contraction} = (1+2) - 1/(1+1) = 1$$
$$\text{Expansion} = 1 - 2/(2+1) = -0.33$$

All orthology groups had values between -0.5 and 2 except for orthogroup BBL000352 (contraction value = -0.66). However, given the low number of group members (eight members from chicken and lizard and nine members from *X. laevis*) we cannot rule out that this is due to the incompleteness of our dataset and not a *bona fide* contraction.

microRNA prediction

We used miRDeep2 [16] (v2.0.0.8 - Marc Friedlander and Sebastian Mackowiak, *personal communication*) in order to predict genomic loci expressing miRNA precursors. Genome assembly Pw_v4.2 was split into 32 files and each indexed using Bowtie (v1.1.2). All FASTQ files from the ten sequenced small RNA libraries were merged into one single file (288.01 million reads) and mapped to each of the indexed genome files using mapper.pl as follows:

```
mapper.pl ALL.FASTQ -e -j -k TGGAATTCTCGGGTGCCAAGG -h -l 18 -m -p PwXX
-s ALL.fa -t ALL_vs_PwXX.arf -v -n
```

The resulting 32 *.arf files were used to predict precursors using miRDeep2.pl as follows:

```
miRDeep2.pl ALL.fa PwXX.fa ALL_vs_PwXX.arf none XENOPUS_MATURE.fasta
none -v 2>ALL_vs_PwXX.report.log
```

Per miRDeep2 recommendations, we included a file of miRBase mature miRNAs from a related species. The file XENOPUS_MATURE.fasta included 178 mature miRNAs from *Xenopus tropicalis* and 21 from *Xenopus laevis*.

miRDeep2 predicted 23,425 precursors. To determine which cutoff minimizes false positives we ran miRDeep2 against the *Xenopus tropicalis* and zebrafish genomes using published small RNAseq data.

The same procedure was applied to the Trinity *de novo* assembled transcriptome Pw_v3.1 to detect primary miRNA transcripts.

When testing miRDeep2 with *Xenopus* and zebrafish datasets, miRDeep2 produced a table with the number of novel of miRNAs reported and known miRNAs detected. As the miRDeep2 score decreases, the rate of novel predictions increases compared to known miRNAs detected. This is a sign of increasing false positives. Based on this calibration we chose a miRDeep2 score of 3 as a cutoff (Supplementary Fig. 3). However, the number of conserved miRNAs predicted in the *P. waltl* genome increased rapidly below a miRDeep2 score of 6. Therefore, we chose score = 6 as a cutoff for genome predicted precursors and a score of 3 for transcriptome predicted precursors. (Supplementary Fig. 3).

RNAseq quantification

Salmon (v0.6.0) [17] was used for all gene expression quantification. An index was generated as follows:

```
salmon index -t Pwaltl.fasta  -i Pwaltl.index --type quasi -k 31
```

RNAseq reads were mapped using the Salmon quasi-mapping algorithm as follows:

```
salmon quant -i Pwaltl.index -l ISR -1 <(gunzip -c
$F/P2024_20XX_R1.fq.gz) -2 <(gunzip -c $F/P2024_20XX_R2.fq.gz) -o
P2024_20XX --geneMap Pwaltl.map
```

Where P2024_20XX is defined in Supplementary Table 3 and Pwaltl.map defines the orthology group that each protein coding transcript belongs to. Results were computed at both transcript and orthology group levels.

Gene expression analysis was performed using DESeq2 within the Trinotate pipeline (https://github.com/trinityrnaseq/trinityrnaseq/wiki/Trinity-Differential-Expression) as follows:

First transcript and gene expression matrices were built as follows

```
perl abundance_estimates_to_matrix.pl --est_method salmon --out_prefix
trans_counts [or group_counts] --name_sample_by_basedir
P2024_2001..P2024_2025
```

Where `P2024_2001..P2024_2025` is the list of transcript level or orthology group level quantifications produced by Salmon.

Next, differential expression analysis was performed at the transcript level using DESeq2 as follows:

```
perl run_DE_analysis.pl --matrix trans_counts.counts.matrix --method
DESeq2 --samples_file samples_described_replicates.txt
```

and at the orthology group level as follows:

```
perl run_DE_analysis.pl --matrix group_counts.counts.matrix --method
DESeq2 --samples_file samples_described_replicates.txt
```

Where `samples_described.txt` defined the replicates for the following samples:

```
lateemb    P2024_2013
lateemb    P2024_2020
larvae     P2024_2022
larvae     P2024_2005
forelimb   P2024_2011
forelimb   P2024_2015
forelimb   P2024_2016
forelimb   P2024_2017
limb3dpa   P2024_2012
limb3dpa   P2024_2018
limb3dpa   P2024_2019
limb7dpa   P2024_2014
limb7dpa   P2024_2024
limb7dpa   P2024_2025
```

Finally, differentially expressed miRNA primary transcripts were extracted from the transcript-level dataset as follows:

```
perl analyze_diff_expr.pl --matrix trans_counts.TMM.EXPR.matrix -P 1e-
2 -C 1
```

Whereas differentially expressed orthology groups were extracted from the group-level dataset as follows:

```
perl analyze_diff_expr.pl --matrix group_counts.TMM.EXPR.matrix -P 1e-
2 -C 1.
```

Supplementary References:

1 Gallien, C. L. [The karyotype of the urodele Pleurodeles poireti Gervais. Comparative study of karyotypes in the genus Pleurodeles]. *Comptes rendus hebdomadaires des seances de l'Academie des sciences. Serie D: Sciences naturelles* **262**, 122-125 (1966).

2 Looso, M. *et al.* A de novo assembly of the newt transcriptome combined with proteomic validation identifies new protein families expressed during tissue regeneration. *Genome biology* **14**, R16, doi:10.1186/gb-2013-14-2-r16 (2013).

3 Abdullayev, I., Kirkham, M., Bjorklund, A. K., Simon, A. & Sandberg, R. A reference transcriptome and inferred proteome for the salamander Notophthalmus viridescens. *Experimental cell research* **319**, 1187-1197, doi:10.1016/j.yexcr.2013.02.013 (2013).

4 Vaz, C. *et al.* Deep sequencing of small RNA facilitates tissue and sex associated microRNA discovery in zebrafish. *BMC genomics* **16**, 950, doi:10.1186/s12864-015-2135-7 (2015).

5 Harding, J. L. *et al.* Small RNA profiling of Xenopus embryos reveals novel miRNAs and a new class of small RNAs derived from intronic transposable elements. *Genome research* **24**, 96-106, doi:10.1101/gr.144469.112 (2014).

6 Faunes, F. *et al.* Expression of transposable elements in neural tissues during Xenopus development. *PloS one* **6**, e22569, doi:10.1371/journal.pone.0022569 (2011).

7 Armisen, J., Gilchrist, M. J., Wilczynska, A., Standart, N. & Miska, E. A. Abundant and dynamically expressed miRNAs, piRNAs, and other small RNAs in the vertebrate Xenopus tropicalis. *Genome research* **19**, 1766-1775, doi:10.1101/gr.093054.109 (2009).

8 Magoc, T. & Salzberg, S. L. FLASH: fast length adjustment of short reads to improve genome assemblies. *Bioinformatics* **27**, 2957-2963, doi:10.1093/bioinformatics/btr507 (2011).

9 Li, H. Exploring single-sample SNP and INDEL calling with whole-genome de novo assembly. *Bioinformatics* **28**, 1838-1844, doi:10.1093/bioinformatics/bts280 (2012).

10 Myers, E. W. The fragment assembly string graph. *Bioinformatics* **21 Suppl 2**, ii79-85, doi:10.1093/bioinformatics/bti1114 (2005).

11 Simpson, J. T. *et al.* ABySS: a parallel assembler for short read sequence data. *Genome research* **19**, 1117-1123, doi:10.1101/gr.089532.108 (2009).

12 Robertson, G. *et al.* De novo assembly and analysis of RNA-seq data. *Nature methods* **7**, 909-912, doi:10.1038/nmeth.1517 (2010).

13 Tamazian, G. *et al.* Chromosomer: a reference-based genome arrangement tool for producing draft chromosome sequences. *Gigascience* **5**, 38, doi:10.1186/s13742-016-0141-6 (2016).

14 Bryant, D. M. *et al.* A Tissue-Mapped Axolotl De Novo Transcriptome Enables Identification of Limb Regeneration Factors. *Cell reports* **18**, 762-776, doi:10.1016/j.celrep.2016.12.063 (2017).

15 Davidson, N. M. & Oshlack, A. Corset: enabling differential gene expression analysis for de novo assembled transcriptomes. *Genome biology* **15**, 410, doi:10.1186/s13059-014-0410-6 (2014).

16 Friedlander, M. R., Mackowiak, S. D., Li, N., Chen, W. & Rajewsky, N. miRDeep2 accurately identifies known and hundreds of novel microRNA genes in seven animal clades. *Nucleic acids research* **40**, 37-52, doi:10.1093/nar/gkr688 (2012).

17 Patro, R., Duggal, G., Love, M. I., Irizarry, R. A. & Kingsford, C. Salmon provides fast and bias-aware quantification of transcript expression. *Nature methods*, doi:10.1038/nmeth.4197 (2017).
