# Supplementary material for: Reading and editing the Pleurodeles waltl genome reveals novel features of tetrapod regeneration
Source: Nat Commun. 2017 Dec 22;8:2286. doi: 10.1038/s41467-017-01964-9 (PMC5741667; doi:10.1038/s41467-017-01964-9)
Supplement: Supplementary file 2 — Description of Additional Supplementary Files [file 41467_2017_1964_MOESM2_ESM.pdf]

Description of Additional Supplementary Files

File Name: Supplementary Movie 1
Description: P. waltl Pax3 CRISPR/Cas9 mosaic knockout larva with muscle-less forelimb (right).

File Name: Supplementary Data 1
Description: Orthology groups. Sheet1: Groups are prefixed with BBL and the number of members from each species is indicated (hsa = *H. sapiens*, mmu = *M. musculus*, gga = *G. gallus*, aca = *A. carolinensis*, xla = *X. laevis*, xtr = *X. tropicalis,* dre = *D. rerio*, ame = *A. mexicanum*, nvi = *N. viridescens*, pwaT = *P. waltl* transcript and pwaG = *P. waltl* gene model). Groups are annotated based on the most frequent gene symbol appearing in the group. Groups with non-salamander members and no annotation are designated unknown-conserved. Groups with only salamander members and no detected Pfam domains are designated unknown-salamander. Note that columns nvi, ame and pwaT are based on transcriptome predicted proteins. All other columns including pwaG are based on gene-model predicted proteins. Sheet 2: The 19,903 groups including a *P. waltl* orthologs and their classification.

File Name: Supplementary Data 2
Description: Conversion table for TransDecoder and Maker to OrthoMCL compatible id headers (hsa = *H. sapiens*, mmu = *M. musculus*, gga = *G. gallus*, aca = *A. carolinensis*, xla = *X. laevis*, xtr = *X. tropicalis,* dre = *D. rerio*, ame = *A. mexicanum*, nvi = *N. viridescens*)

File Name: Supplementary Data 3
Description: Conversion table for TransDecoder and Maker to OrthoMCL compatible id headers (pwa = *P. waltl*)

File Name: Supplementary Data 4
Description: *P. waltl* miRNAs. miRDeep2 calibration runs with *Xenopus* and zebrafish datasets, genome predictions, transcriptome predictions, mature miRNA quantification and the top 10 miRNAs upregulated in the regenerating tissue (limbs 3dpa and 7dpa) compared to the uninjured adult tissue (brain, eyes, heart, liver, lung).
